# The role of gut microbiota–mitochondria crosstalk in neurodegeneration: Underlying mechanisms and potential therapies

**DOI:** 10.4103/NRR.NRR-D-24-01419

**Published:** 2025-04-29

**Authors:** Tianjuan Ju, Yaoyuan Zhang, Lipeng Liu, Xitong Zhao, Xinwei Li, Changfeng Liu, Shukai Sun, Li-an Wu

**Affiliations:** State Key Laboratory of Oral & Maxillofacial Reconstruction and Regeneration, National Clinical Research Center for Oral Diseases, Shaanxi Clinical Research Center for Oral Diseases, Department of Pediatric Dentistry, School of Stomatology, The Fourth Military Medical University, Xi’an, Shaanxi Province, China

**Keywords:** Alzheimer’s disease, amyotrophic lateral sclerosis, gut microbiota, gut‒brain axis, microbiota‒mitochondria crosstalk, neurodegenerative diseases, neuroinflammation, Parkinson’s disease, probiotic, short chain fatty acid

## Abstract

Emerging evidence suggests that the gut microbiota is closely associated with the pathological manifestations of multiple neurodegenerative diseases via the gut-brain axis, which refers to the crosstalk between the gut and the central nervous system. More importantly, mitochondria have been considered prominent mediators of the interplay between the gut microbiota and the brain. Intestinal microbes may modulate mitochondrial function in the central nervous system to affect the progression of neurodegenerative diseases. Mitochondria are essential for meeting the host’s substantial neuronal metabolic demands, maintaining excitability, and facilitating synaptic transmission. Dysfunctional mitochondria are considered critical hallmarks of various neurodegenerative diseases. Therefore, this review provides novel insights into the intricate roles of gut microbiota-mitochondrial crosstalk in the underlying mechanisms during the progression of neurodegeneration, as well as the existing potential therapeutic strategies for neurodegenerative disorders. These suggest intestinal microbiota-mitochondrial interaction play a crucial role in the occurrence and development of neurodegenerative diseases, and targeting this interaction may be a promising therapeutic approach to neurodegenerative diseases. However, this review found that there was relatively little research on the effect of this crosstalk on other neurodegenerative diseases, such as Huntington’s disease and Multiple sclerosis, and the potential therapeutic strategies were translated into clinical trials, which face many challenges in developing personalized treatment plans based on the unique gut microbiota of different individuals.

## Introduction

The gut microbiome is the collective term used to describe the bacteria, archaea, viruses, and other microorganisms residing in the human gastrointestinal tract. These bacteria participate in defensive strategies and metabolic processes and exert profound effects on the physiological status of the human body in both health and disease (Kesika et al., 2021). Recent research advancements have revealed a robust link between the gut microbiota, neuroinflammation and neurodegeneration. This finding indicates that the gut microbiota not only affects cognitive function and psychiatric disorders but may also precipitate neurodegenerative diseases such as Alzheimer’s disease (AD), Parkinson’s disease (PD), and amyotrophic lateral sclerosis (ALS) via the microbiota–gut–brain axis, thus indicating the essential bidirectional communication between the gut microbiota and the central nervous system (CNS) (Mou et al., 2022). Further investigation is now needed to fully comprehend the fundamental processes that regulate the communication channels through which gut microbes interact with the brain. These systems are linked to brain activation, immune system regulation, and the endocrine pathway within the gut.

Mitochondria are complex and dynamic organelles that maintain cellular health and homeostasis by executing various functions via a range of ongoing processes, including biogenesis, mitophagy, fission, and fusion (Yang et al., 2024). Mitochondria are the principal producers of adenosine 5′-triphosphate (ATP) and function as the source of cellular energy under physiological conditions (Rowland and Voeltz, 2012; Trigo et al., 2022). The CNS is the most energy-dependent system. The regulation of calcium homeostasis and the facilitation of antioxidant defense signaling in the CNS depend on the production of ATP by mitochondria. The cytoplasmic volume of neuronal cells is known to be comprised of 40% mitochondria thus reflecting the dependence of neuronal survival and excitability upon energy production. Consequently, mitochondrial dysfunction presents a significant challenge to the brain. Mutations in mitochondrial DNA (mtDNA) and genes, alterations in mitochondrial dynamics (fusion/fission, movement, size, morphology, and transport), and impaired transcription are the etiologies of mitochondrial dysfunction, leading to bioenergetic defects (Golpich et al., 2017). A significant influx of calcium, and the overproduction of reactive oxygen species (ROS) and active nitrogen by impaired mitochondria can lead to the formation of mitochondrial permeability transition pores which are known to be associated with neurodegenerative diseases (Li et al., 2017). Furthermore, several disease-specific proteins, such as amyloid-β (Aβ) plaques deposits, tau deposition and α-synuclein (α-syn) aggregation, associated with genetic variations of neurodegenerative diseases exhibit significant interactions with impaired mitochondria. Therefore, mitochondrial dysfunction has been recognized as a prominent feature in the pathogenesis of neurodegenerative disorders (Schon and Przedborski, 2011; Rajkumar et al., 2020; Rani and Mondal, 2020; Theunissen et al., 2021).

**Figure 1:**
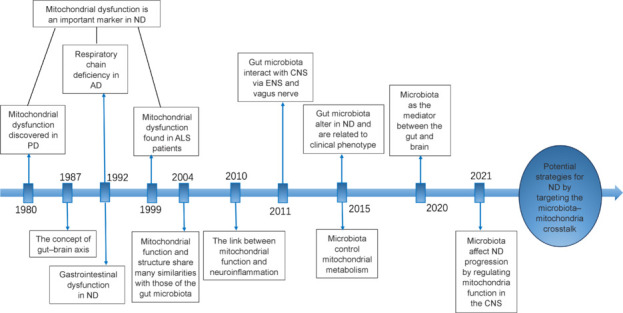
Timeline showing mitochondria as an important mediator in the gut–brain axis to regulate the progression of NDs. AD: Alzheimer’s disease; ALS: amyotrophic lateral slierosis; CNS: central nervous system; ENS: enteric nervous system; ND: neurodegenerative disease; PD: Parkinson’s disease.

A new and exciting field of study is interaction between the gut microbiota and the host mitochondria in both healthy and sick states. Given that mitochondria originated in a strain of Proteobacteria, the characteristics shared by both prokaryotic and eukaryotic mitochondria are fundamental to their shared function and structure with the gut microbiota (Dyall et al., 2004; Lucattini et al., 2004). Consequently, microbes in the intestines can target the mitochondria of host cells and play a pivotal role in the induction of immune responses. Evidence suggests that signaling between the gut microbiota and mitochondria can alter functionality of the epithelial barrier, activate immune cells, alter mitochondrial metabolism, and induce inflammasome signaling (Jackson and Theiss, 2020). Numerous diseases, including colorectal cancer, diabetes, obesity, and chronic intestinal inflammation, are strongly linked to mitochondrial dysfunction caused by gut microbiota (Jackson and Theiss, 2020; Vezza et al., 2020; Colangeli et al., 2023). Traditionally, it was generally considered that the CNS was relatively impermeable to gut bacteria because of the blood–brain barrier, and that this structure impacted the progression and incidence of neurological illnesses. However, recent studies have shown that mitochondria play a mediating role in the gut-brain axis, a communication link between the gut microbiota and mitochondria in the CNS that affects the development of neurodegenerative disorders (**Figure 1**). To date, there have been only a few reviews on this subject, focusing on subjects such as the relationships between mitochondria and microbiota, the effects of metabolites produced by gut microbes on neurodegenerative diseases, and the interplay between the homeostasis of intestinal microbes and mitochondrial energy metabolism (Kramer, 2021; Borbolis et al., 2023; Qiao et al., 2024). In contrast, this review more comprehensively summarized the various gut microbiota-derivates that regulate mitochondrial function in the CNS, and focused on the effects of gut microbiota-mitochondrial interaction on neurodegenerative diseases, explained from the new perspective of mitochondrial regulatory mechanism and neuroinflammation. In addition, emerging techniques targeting the microbiota-mitochondria interaction were reviewed, possible future research directions and the challenges in clinical translation were elaborated.

## Literature Search Strategy

A literature search of the PubMed database spanning from 2013 to 2024 was conducted. The initial selection of articles for evaluation used the following keyword combinations: neurodegenerative disease, gut–brain axis, gut microbiota, short-chain fatty acids, secondary bile acids, H_2_S, indole-3-propionic acid, urolithin A, neurotransmitters, hormones, and mitochondria. The authors conducted a selection of relevant studies to identify potentially valuable research, initially screening titles, abstracts, and full texts utilizing specific keywords. Only studies that focused on the gut microbiota or their metabolites in connection to mitochondrial function in the CNS were included in the evaluation of the study on the role of gut microbiota-derived metabolites in neurodegeneration. This was done so that the research could be evaluated. Neither the language nor the type of study was prohibited.

## Mitochondrial Dysfunction and Neurodegeneration

### Mitochondrial dysfunction as a significant marker of neurodegeneration

Neurodegenerative diseases are characterized by a gradual and continuous decline in neuronal status, either structurally or functionally, along with the buildup of proteins inside neurons, ultimately undermining the functionality of the nervous system (Lin and Beal, 2006). Accumulating evidence supports the fact that mitochondrial dysfunction represents a foundational cause and crucial hallmark of various neurodegenerative diseases. Conditions such as ALS, PD, AD, multiple sclerosis, and Huntington’s disease are all significantly impacted by this dysfunction. Furthermore, it is essential to recognize that mitochondrial dysfunction does not occur in isolation; rather, it is influenced by a multitude of factors that may include genetic predispositions, environmental triggers, and metabolic changes. These insights underline the complexity of neurodegenerative diseases and the critical role that mitochondrial health plays in their progression and manifestation (Klemmensen et al., 2024). In this section, we explain the influence of mitochondrial dysfunction on the onset and progression of ALS, PD, and AD.

The exact etiology of AD, the most common form of neurodegeneration, remains unclear. The amyloid hypothesis is regarded by most researchers as the most compelling explanation for the etiology of AD. Nonetheless, mitochondrial dysfunction and other mechanisms may also be involved. The mitochondrial cascade theory suggests that most instances of AD begin with mitochondrial malfunction, with other clinical symptoms considered as subsequent consequences, as recently stated by several studies (Swerdlow et al., 2010; Swerdlow, 2018). The onset of AD is thought to be considerably affected by mitochondrial malfunction (Wang et al., 2020). Numerous tests have revealed substantial mitochondrial abnormalities in the brains of patients suffering from AD (Swerdlow et al., 2010). Mitochondrial failure can lead to intracellular hyperphosphorylated tau neurofibrillary tangles and Aβ plaques, the principal neuropathological characteristics of AD (Selkoe, 2001). Aβ plaques and tau deposits may arise from mitochondrial malfunction (Monzio Compagnoni et al., 2020). Oxidative stress can cause damage before the development of Aβ plaques and the deposition of tau, whereas mitochondrial damage represents the primary event that occurs prior to neurodegenerative disorders (Olagunju et al., 2023). Mice deficient in mtDNA polymerase γ or treated with complex IV inhibitors have been shown to lead to the elevated accumulation of Aβ_42_ and amyloid plaques, along with tau-positive staining particles in the frontal cortex (Szabados et al., 2004; Kukreja et al., 2014). The aggregation of Aβ plaques and the accumulation of tau have a complex impact on mitochondrial function. Aβ induces depolarization of the mitochondrial membrane, reduces oxygen consumption, and disturbs the balance between mitochondrial fusion and fission (Pereira et al., 1998; Wang et al., 2009). Furthermore, these events can specifically inhibit the respiratory chain, which includes complexes I, III and IV. Mutations associated with tau disease have been shown to modify the structure, redox state, and dynamics of mitochondria (Perbet et al., 2023).

PD is the second most common neurodegenerative disorder and is marked by the gradual degeneration of dopaminergic neurons in the substantia nigra of the midbrain (Corrêa et al., 2025). Factors that increase the likelihood of developing PD, such as environmental influences, genetic predispositions, and biological elements, lead to the disorder via the disruption of mitochondrial function (Zanon et al., 2018). Mitochondria make a significant contribution to PD, as evidenced by comprehensive genetic and biochemical evidence (Schapira, 2008; Rango and Bresolin, 2018). Specific causative genes for familial PD have been identified as being directly associated with mitochondrial biology. Prominent instances include PARKIN and PTEN-induced putative kinase 1 (PINK1), which participate in mitochondrial autophagy mechanisms; pathogenic mutations in PARKIN and PINK1 are known to account for the autosomal recessive transmission of early-onset PD (Kitada et al., 1998; Valente et al., 2004). Notwithstanding the limitations described earlier, the administration of mitochondrial toxins, particularly 1-methyl-4-phenyl-l,2,5,6-tetrahydropyridine (MPTP) and rotenone has been shown to induce the clinical and neuropathological features of PD in both human and animal models (Zhang et al., 2024). Additional mitochondrial mechanisms associated with PD encompass compromised respiratory chain activity, particularly complex I, and alterations in mitochondrial DNA (Monzio Compagnoni et al., 2020). PD is characterized neuropathologically by the accumulation of intracellular α-syn proteins (Capriotti and Terzakis, 2016), which also lead to mitochondrial dysfunction. The accumulation of α-syn alters mitochondrial dynamics and mitophagy, induces membrane permeabilization and directly exacerbates damage by elevating the formation of ROS and by precipitating a bioenergetic crisis. Conversely, faulty mitochondria can promote pathogenic aggregation (Grünewald et al., 2019; Kamienieva et al., 2021; Rybarski et al., 2023).

The defining characteristic of ALS, a grave disorder, is the selective destruction of upper and lower motor neurons. This condition affects the pyramidal tract, the anterior horn cells of the spinal cord, and the motor nuclei of the brainstem. Regrettably, the etiology and pathogenesis of ALS have yet to be fully elucidated (Devi et al., 2008). Numerous pathophysiological changes in ALS are associated with mitochondrial dysfunction, protein misfolding, and epigenomic mutations. Mitochondrial involvement has been recognized in numerous models of ALS and cases associated with known pathogenic gene mutations. Dark mitochondrial aggregates have been identified in motor neurons of the anterior horn in ALS patients, and abnormal mitochondrial ultrastructures have been detected in autopsy/biopsy specimens of spinal cord and muscle (Sasaki and Iwata, 1996). In primary neuron cultures derived from animal models of ALS, alterations in mitochondria morphology and impaired mitochondrial transport along axons have been noted (Magrané et al., 2012; Wang et al., 2013). The abnormal buildup of altered proteins inside or on the surface of mitochondria frequently leads to mitochondrial impairment in models of ALS (Magrané et al., 2014; Pickles et al., 2016). Ultimately, epigenetic occurrences, including gene mutations linked to ALS, can impair protein homeostasis, thus leading to protein misfolding and mitochondrial dysfunction. The interplay between protein misfolding and mitochondrial dysfunction can influence the advancement of ALS.

Existing literature strongly supports the fact that mitochondrial damage is a crucial factor in the progression and persistence of neurodegenerative disorders, irrespective of their initial etiology. Pathological protein deposits and mitochondrial dysfunction may expedite neurodegeneration, thus accelerating neuronal death; this detrimental loop could underlie disease progression. Novel pharmaceutical strategies are now being investigated and developed due to the significant role of mitochondria in neurodegenerative disorders.

### Mitochondrial dysfunction induces neuroinflammation during neurodegeneration

The synthesis of chemokines, proinflammatory cytokines (e.g., interleukin-1β (IL-1β), IL-6, IL-18 and tumor necrosis factor-α (TNF-α)), small-molecule mediators (e.g., prostaglandins and nitric oxide (NO), and ROS by innate immune cells within the CNS are characteristic of neuroinflammation (DiSabato et al., 2016). In the realm of neurodegenerative diseases, neuroinflammation is typically a persistent process that does not self-resolve and is regarded as a significant contributor to the disease (Han et al., 2024). The release of proinflammatory molecules and chemotaxis can contribute to neuronal death, synaptic dysfunction and the inhibition of neurogenesis (Lyman et al., 2014). Furthermore, released chemokines promote microglia-induced neuroinflammation, thus provoking local inflammation (Lyman et al., 2014). Once astrocytes and microglia have been activated, they further promote the deposition of pathological proteins. These types of proteins, such as Aβ, also activate microglia and induce neuronal dysfunction (Lian et al., 2015, 2016). In summary, neuroinflammation is a prominent pathology in neuroinflammation caused by microglia, astrocytes, neurons and other cells in a synchronized manner.

**Figure 2:**
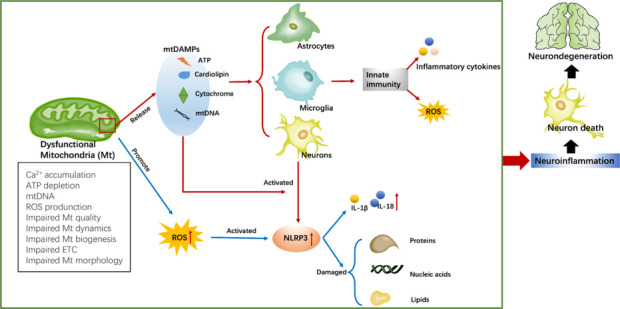
The process by which mitochondrial dysfunction induces the neuroinflammation involved in neurodegenerative diseases. Impaired mitochondria release mtDAMPs into the cytoplasm or extracellular environment; these mtDAMPs are recognized by the receptors of astrocytes, microglia, and neurons, ultimately causing neuroinflammation by activating the innate immune system or the NLRP3 complex. The overproduction of mtROS also activates the NLRP3 complex to promote the expression of proinflammatory cytokines and damage proteins, nucleic acids, and lipids in the brain. Created with Procreat 5.3.6 and Microsoft PowerPoint. ATP: Adenosine triphosphate; ETC: electron transport chain; IL-18/1β: interleukin-18/1β; mtDNA: mitochondrial DNA; mtDAMPs: mitochondrial damage-associated molecular patterns; NLRP3: NLR family, pyrin domain containing protein 3; ROS: reactive oxygen species.

An increasing number of studies indicate that mitochondrial dysfunction serves as a signaling hub that initiates neuroinflammation (**Figure 2**; Litwiniuk et al., 2021; Galizzi and Di Carlo, 2023). When pathological proteins disrupt mitochondrial function, microglia and astrocyte receptors recognize mitochondrial damage-associated molecular patterns, or mitochondrial damage-associated molecular patterns (mtDAMPs). An oxidative and neuroinflammatory milieu is established when this identification triggers an innate immune response, which in turn produces proinflammatory cytokines and ROS (Hansson Petersen et al., 2008; Cardoso and Empadinhas, 2018; Klionsky et al., 2021). When mitophagy fails to remove highly damaged mitochondria in an appropriate manner, mtDNA, cytochrome c, cardiolipin, and ATP are released into the cytoplasm and the extracellular environment (Deus et al., 2022). Cardiolipin, mtDNA, cytochrome c, and ATP are the main components of mtDAMPs. According to recent research, neurodegenerative illnesses can cause behavioral symptoms in mice when faulty mtDNA is injected into their brains, including motor, cognitive, and neuropsychiatric abnormalities. The mtDNA-induced activation of Toll-like receptors (TLRs) 4 and 9 pathways in neurons can result in increased levels of oxidative stress and neuronal cell death (Tresse et al., 2023). In addition, neuroinflammation in AD can be exacerbated when mtDNA triggers the microglia to produce inflammatory mediators, such as TNF-α (Wilkins et al., 2015). Furthermore, mtDNA has the ability to upregulate the stimulator of interferon genes (STING)-NLR family, pyrin domain containing protein 3 (NLRP3)-IL-1β pathway, which facilitates the recruitment and infiltration of neutrophils and can exert impact on the advancement of AD (Xia et al., 2024). Cytochrome c is released into the cytoplasm in the form of mtDAMPs following mitochondrial damage. Neuronal death can occur when cytochrome c activates the microglia and astrocytes via TLRs, thus leading to the secretion of inflammatory mediators such as IL-1β and granulocyte-macrophage colony-stimulating factor (GM-CSF) (Wenzel et al., 2019). The process begins with mitochondrial fragmentation and ends with the production of IL-1β via activation of the neural inflammasome NLRP3 and the subsequent release of cardiolipin (Silva et al., 2020a). Furthermore, cardiolipin may contribute to the progression of neurodegenerative illnesses by modulating the phagocytic activity of glial cells and the release of inflammatory cytokines and cytotoxins in a manner that is reliant on TLR4 (Wenzel et al., 2021, 2023). The purinergic receptor family, particularly the P2X7 and P2X4 receptors, recognizes the release of ATP from mtDAMPs into the extracellular environment. In PD, P2X4 activation may initiate NLRP3-mediated neuroinflammation and dopaminergic neuron loss (Wang et al., 2022b). The release of inflammatory mediators, such as TNF-α, from microglia, and the induction of neuronal endoplasmic reticulum stress by the activation of P2X7 may exacerbate the progression of ALS (Bartlett et al., 2022). The release of molecules from mtDAMPs can be affected by oxidative damage that arises from mitochondrial malfunction (Silva et al., 2022). Furthermore, inflammatory chemicals secreted from glial cells can modify and impact mitochondrial activity in a negative manner. These processes can give rise to apoptosis and mitochondrial malfunction, which in turn can accelerate the progression of several neurodegenerative diseases (Jetto et al., 2022). Therefore, it is evident that the development of neurodegenerative diseases can be influenced by the interplay between chronic inflammation and mitochondrial oxidative stress.

## The Gut Microbiota Is Closely Related to Neurodegenerative Diseases

The human gut harbors more than 100 trillion bacteria, which express approximately 150-fold more genes than in the human genome (Ley et al., 2006). Recent data suggests that the gut microbiota play a role in the development of neurodegenerative diseases via the “microbiota-gut–brain axis” (Hu et al., 2016; Xie et al., 2022). Certain facets of neuronal function are intimately associated with the gut microbiota, including neuroinflammation, myelination, modulation of the blood–brain barrier integrity, and abnormal protein build-up (Seo and Holtzman, 2020). Recent research on the vagus nerve yielded encouraging findings relating to the the relationship among gut microorganisms, the brain, and neurodegenerative illnesses. The development of neuropathic disease can be initiated or regulated by microbial metabolites that infiltrate the CNS via the vagus nerve. Commensal bacteria can mediate the creation and release of neuroactive substances, such as hormones and neurotransmitters. These chemicals can influence signals from the vagus nerve to the brain and the functionality of the enteric nervous system (Parker et al., 2020; Leeuwendaal et al., 2021). Furthermore, alterations in the gut microbiota and bacterial metabolites may result in enhanced permeability of the blood–brain and intestinal barriers. Consequently, substances from the gut microbiota, including short-chain fatty acids (SCFAs), accumulate in the brain and can either induce or mitigate neuroinflammation within the microenvironment of the brain. This establishes a foundation for the pathological development of neurodegenerative disorders (Roy Sarkar and Banerjee, 2019). Pathogenic modifications may arise when the concentrations of microbiome metabolites increase in the blood, thereby amplifying the production of immune cells or peripheral immune-proinflammatory cytokines (Fung et al., 2017; Li et al., 2021). Microbes in the gut can exert significant influence on the progression of neurodegenerative illnesses via these pathways, and disturbances in the intestinal microbiota may be pivotal in the onset of these conditions (Dinan and Cryan, 2017a).

Previous research indicates that patients with AD exhibit significantly diminished fecal microbial diversity, characterized by a significantly lower proportion of *Firmicutes* compared to the healthy population (Kim et al., 2020b). Proinflammatory bacteria and their derivative lipopolysaccharide (LPS) in the gut microbiota are significantly associated with brain amyloids, peripheral inflammation, and cognitive decline in AD patients (Cattaneo et al., 2017). The combination of *Lactobacillus mucosae NK41* and *Bifidobacterium longum NK46* probiotics was shown to inhibit gut bacteria that generated LPS and suppressed NF-κB-mediated brain-derived neurotrophic factor (BDNF) production, thereby mitigating cognitive deficits and neuroinflammation in a rat model of AD (Ma et al., 2023). Aβ, a pathogenic protein associated with AD, accumulates in the brain as a result of intestinal epithelial barrier dysfunction and vascular Aβ deposition. Gut microbial dysbiosis may result in the formation of amyloid-containing biofilms, thereby expediting Aβ accumulation (Honarpisheh et al., 2020; Miller et al., 2021). Recent research indicated that alterations in the composition of the gut microbiome were linked to tau and Aβ pathology, but not to neurodegenerative biomarkers, thus suggesting that changes in intestinal microecology occur early in this condition and may serve as an indicator of preclinical AD (Ferreiro et al., 2023). In the absence of gut microbiota, Aβ pathologies are reduced in Aβ precursor protein transgenic mice (Harach et al., 2017). The gut microbiota can influence the response to Aβ amyloidosis by modulating astrocytes via microglial-dependent and independent pathways (Chandra et al., 2023). Apolipoprotein E, a prevalent genetic risk factor for AD, has been shown to influence the structure and function of gut microbiota in both humans and mice, along with the expression of microbiota-associated amino acids and SCFAs (Tran et al., 2019). Similarly, alterations in the abundance of gut microbiota have been confirmed in patients with PD; the composition exhibited a notable prevalence of *Akkermansia*, *Lachnospiraceae*, *Verrucomicrobiaceae*, and *Ruminococcaceae*, alongside a reduction in the concentrations of SCFAs (Scheperjans et al., 2015; Unger et al., 2016; Heravi et al., 2023). A previous study that overexpressed α-syn in mice showed that the gut microbiota are essential for microglial activation, motor deficits, and α-syn aggregation. Studies have demonstrated that the gut microbiota can influence movement disorders in animals, and that alterations in the human intestinal microbiota represent a risk for PD (Sampson et al., 2016). Furthermore, the parasympathetic nerves and enteric nervous system are known to be most affected by α-syn pathology. Thus, the emergence of gastrointestinal dysfunction and intestinal lesions occurs prior to the development of early motor impairment in PD patients (Scheperjans et al., 2015). The involvement of gut microbiota in the advancement of PD via the vagus nerve has generated considerable research interest. The prevalence of PD in individuals with peptic ulcers who have undergone vagotomies diminishes with age and may also postpone the onset of this disease (Svensson et al., 2015; Killinger et al., 2018). Moreover, the gut microbiota may significantly influence the complexity of ALS development (Boddy et al., 2021). Research has revealed that the composition of gut microbiota in individuals with ALS differs from that of healthy people, marked by a reduction in Firmicutes, Megamonas, and Prevotella, as well as an increase in proinflammatory mediators in fecal matter (Rowin et al., 2017; Nicholson et al., 2021; Özaydin Aksun et al., 2024). In murine models of ALS, dysbiosis of local gut microbiota was associated with disease severity. Researchers found that levels of niacinamide (NAM) in the systemic and cerebrospinal fluids of ALS patients were diminished, and that the introduction of these microbial metabolites may exacerbate the symptoms; furthermore, supplemental NAM may enhance mobility in ALS mouse models (Blacher et al., 2019). In other research, butyric acid supplementation was shown to significantly diminish the aggregation of SOD1^G93A^ protein in the intestine, restore gut microbiota equilibrium, and prolong the lifespan of mice of ALS (Zhang et al., 2017).

**Figure 3:**
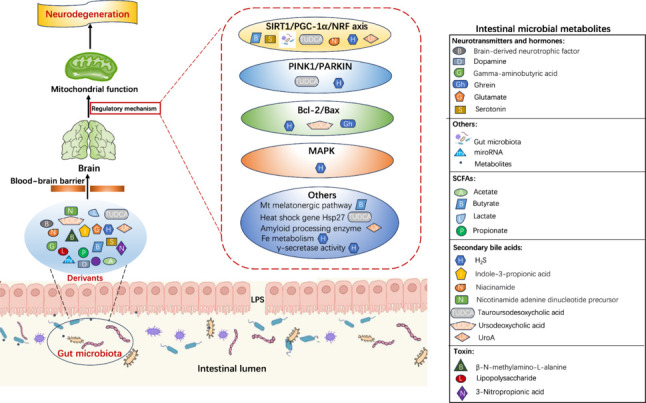
The signaling pathways of gut microbiota-derived metabolites regulating mitochondria function in the central nervous system during neurodegenerative diseases. Some metabolites regulate mitochondrial function in the central nervous system by regulating the activity of SIRT1/PGC-1α/NRF, PINK1/PARKIN, Bcl-2/Bax, MAPK and other signaling pathways or molecules. Created with Procreat 5.3.6 and Microsoft PowerPoint. Bcl-2/Bax: B-cell lymphoma-2/Bcl-2-associated X protein; LPS: lipopolysaccharide; MAPK: mitogen-activated protein kinase; Mt: mitochondria; PINK1/PARKIN: PTEN-induced putative kinase 1/parkin; SIRT1/PGC-1α/NRF: silent mating type information regulation 2 homolog-1/peroxisome proliferator-activated receptor γ coactivator 1 alpha/nuclear factor erythroid 2-related factor.

Overall, the development of neurodegenerative disease and clinical symptoms are associated with alterations in the gut flora. Some scientists believe that neurodegenerative diseases could spread to the CNS. Evidence from these sources indicates that the early motor symptoms of PD appear in the gastrointestinal system first, that the transmission of α-syn from the gut to the brain is certainly possible, and that Aβ accumulates in the brain after vascular Aβ deposition and disruption to the intestinal epithelial barrier. On the contrary, recent research has shown that information can actually travel in the opposite direction, from the brain to the gut (Vanden Berghe, 2023). Patients with neurodegenerative diseases frequently exhibit increased intestinal inflammation, and this correlation may help us to elucidate the specific mechanisms that underlie these observations (Xiromerisiou et al., 2023). However, further research is now needed to elucidate the temporal and causal relationships between neurodegeneration and the intestinal microbiota, and the suitability of these microbes as biomarkers. A multitude of experts are now investigating interactions between the gut flora and the brain. An imbalance in the gut microbiota and vagal transmission may result in intestinal inflammation, subsequently leading to the development of neurodegenerative diseases (Rani and Mondal, 2021). According to a recent study, mitochondria play a significant mediating role in the gut microbiota-brain axis, which can exert influence on neurological conditions. Intestinal microbiome-derived metabolites or other bioactive molecules can regulate the mitochondrial function of the brain, thereby affecting the neuronal function of the CNS and the development of neuroinflammation during the progression of neurodegenerative diseases. Therefore, it is important for us to consider the derivates of the gut microbiota that can modulate mitochondrial function in the brain, including microbiome-derived or regulated metabolites, neurotransmitters, hormones and microRNAs (miRNAs) (**Figure 3**), the regulatory mechanism of some derivates on mitochondrial function in the CNS, and the effects of intestinal microbial-mitochondrial interaction on neuroinflammation during neurodegeneration (**Additional Table 1**).

**Additional Table 1:** Summary of the mechanism and effect of derivates from gut microbiota on CNS mitochondria during neurodegenerative disorders

Microbial metabolite		Mitochondria effect	Signaling pathway	Neuronal effect	Neurological disorder	Reference
Gut microbiota		Mitochondrial function; Mitochondrial metabolic network (TCA cycle); ATP production; Mitochondrial specific respiratory chain complex II; Mitochondrial dynamic dysregulation	H3K4me3 and H3K9ac; NLRP3 inflammasome; TLR4	Biologically altered microglia; Related to microglial morphology, proliferation, and activation; Innate immunity; α-syn	ad, pd	Erny and Prinz, 2017; Fan et al., 2020; Mezö et al., 2020; Erny et al., 2021; Magalhäes et al., 2023
Probiotics		Accelerated mitochondrial autophagy	PTEN-induced putative kinase 1 (PINK1)/PARKIN pathway	Improved motor function and longevity	pd	Hawrysh et al., 2023
Short-chain fatty acids	Propionate	Restored mitochondrial function; Normalized mitochondrial morphology		Hindered brain atrophy	als	Haghikia et al., 2015; Duscha et al., 2020
	Butyrate	Improved mitochondria function; Increased mitochondrial respiratory capacity; Improved mitochondrial network; Promoted mitochondrial biogenesis	PGC-1α; NRF1/TFAM axis; Mitochondrial melatonergic pathway	Anti-inflammatory effects of astrocytes; Increased lactate shuttling; Induced ATP transmission; Increased neuronal differentiation ability	ad, als, pd	Uittenbogaard et al., 2018; Li et al., 2022; Wang et al., 2022a; Karbownik et al., 2023
	Lactate	Main source of pyruvate		Substrates for neuronal energy metabolism; Cognitive impairment; Neuroinflammation		Kann, 2024
	Acetate	Restored mitochondrial activity and morphology Increased ROS		Modulated microglial maturation and phagocytosis; Aβ deposition	ad	Erny et al., 2021
Toxins	Lipopolysaccharide	Caused mitochondrial dysfunction; Increased mitochondrial ROS; Impaired mitochondrial fission	NLRP3 inflammasome	Neuroinflammation Neuron death; Innate immunity activation; α-syn oligomerization	pd	Esteves et al., 2023a
	β-N-methylamino-L-alanine	Decreased oxygen consumption rate; Increased ROS; Destroyed mitochondrial structure	Activated neuronal TLRs; NLRP3 inflammasome	Neuroinflammation tau, Aβ and a-syn aggregation; Activated neuronal innate immune response	ad, pd	Silva et al., 2020a; Esteves et al., 2023b; Vanden Berghe, 2023
Secondary bile acids	Ursodeoxycholic acid	Ameliorated neuro–mitochondrial damage	Bcl-2/Bax	Suppression of neuron death and sensorimotor deficits	hd, pd	Keene et al., 2001, 2002; Abdelkader et al., 2016
	Tauroursodeoxycholic acid	Regulated mitochondrial autophagy; Restored mitochondrial structure abnormalities (mitochondrial swelling and loss of mitochondrial crest)	Increased antioxidant enzymes; PINK1/PARKIN pathway; NRF-2 signaling; Inhibited Hsp27	Protected neuroblastoma cells from death; Prevented neurodegeneration; Antioxidant effect	pd	Abdelkader et al., 2016; Fonseca et al., 2017; Moreira et al., 2017; Rosa et al., 2017, 2018; Qi et al., 2021
Hydrogen sulfide		Mitochondria released excessive cytochrome c and ROS; Regulated mitochondrial activity; Maintained mitochondrial membrane potential; Eliminated intracellular ROS accumulation; Protected mtDNA	SIRT1-PGC-1α; PINK1/PARKIN pathway; Bcl-2/Bax; Cytochrome c protein; Mitogen-activated protein kinase pathway; Ferrous metabolism	Aggregation of α-syn oligomers and fibrils; Antioxidant and anti–inflammatory effects; Suppressed neurotoxicity; Promoted energy production and cell viability; Ameliorated memory deficits	pd, ad	Hälldin and Land, 2008; Tang et al., 2010, 2012; Vandiver et al., 2013; Zhao et al., 2016a; Vandini et al., 2019; Tabassum et al., 2020; Murros, 2022; Zhou and Wang, 2023
Indole-3-propionic acid		Increased mitochondrial respiration rate and membrane potential; Reduced mitochondrial ROS production		Excellent antioxidant properties	ad, hd	Jiang et al., 2022
Urolithin A		Reduced mitochondrial calcium inflow and ROS accumulation; Increased mitochondrial aldehyde dehydrogenase activity; Induced mitochondrial autophagy	PINK1/PARKIN NRF-2; NLRP3 inflammasome; SIRT1-PGC-1α	Inhibited oxidative stress of neurons; Improved cognitive function; Inhibited neuroinflammation; Promoted microglial survival	ad, pd	Xu et al., 2018; Kujawska et al., 2019; Kim et al., 2020a; Lee et al., 2021; Liu et al., 2022; Qiu et al., 2022
NAD^+^ precursors		Supported mitochondrial integrity and function; Facilitated mitochondrial fission and autophagy clearance; Accelerated mitochondrial biogenesis	SIRT1-PGC-1α; SIRT1-adenosine AMPK-PGC-1α axis	Reduced glial cell activation; Suppressed motor neuron loss	als, ad, pd	Jackson et al., 1995; Kang and Hwang, 2009; Fang et al., 2017, 2019b; Blacher et al., 2019; Harlan et al., 2020; Wang et al., 2021
Neurotransmitters	Glutamate	Increased mitochondrial respiration and intracellular ROS; Promoted mitochondrial permeability transition pore opening and dissipation of mitochondrial membrane potential		Neuronal cytotoxicity		Dubey Tiwari et al., 2023; Lin et al., 2023
	Serotonin	Increased mitochondrial biogenesis and ATP generation; OXPHOS efficiency and mtDNA	SIRT1-PGC-1α axis	Neuroprotection Increased neuronal survival		Fanibunda et al., 2019; Fanibunda and Vaidya, 2021
	Brain-derived neurotrophic factor	Inhibited mitochondrial dysfunction		Antioxidative stress		Chen et al., 2017b
	Dopamine	Suppressed the respiratory complex I; Depolarized mitochondria		Neuronal cytotoxicity		Brenner-Lavie et al., 2008; Czerniczyniec et al., 2010
	gaba	Promoted NADH production and TCA cycle		Reduction in GABA-energy signaling		Kanellopoulos et al., 2020
Hormones (indirect effect)	Ghrelin Incretin hormone GLP-1	Ameliorated mitochondrial dysfunction; Inhibited mitochondrial depolarization and ROS generation Led to mitochondrial dysfunction	Bcl-2/Bax/Caspase-3 Activated NLRP3	Improved cognitive function; Neuroprotective Neuroinflammation	AD; PD PD	Dong et al., 2009; Martins et al., 2013; Moon et al., 2014; Rees et al., 2023 Yamane and inagaki, 2018; Jackson et al., 2019
MicroRNA (indirect effect)		Promoted the degeneration of hippocampal mitochondria	Downregulated Glu/Asp rich carboxy-terminal domain 2/ serine/threonine kinase 39	Anxiety-like behavior		Li et al., 2023

AD: Alzheimer's disease; ALS: amyotrophic lateral sclerosis; AMPK: adenosine 5'-monophosphate (AMP)-activated protein kinase; ATP: adenosine 5'-triphosphate; Aβ: amyloid-β; GABA: γ-Aminobutyric acid; HD: Huntington's disease; mtDNA: mitochondrial DNA; NAD+: nicotinamide adenine dinucleotide; NLRP3: NLR family, pyrin domain containing protein 3; NRF1: nuclear factor erythroid 2-related factor 1; OXPHOS: oxidative phosphorylation; PD: Parkinson's disease; PGC-1α: peroxisome proliferator-activated receptor γ coactivator 1 alpha; ROS: reactive oxygen species; SIRT1: silent mating type information regulation 2 homolog-1; TCA: tricarboxylic acid; TFAM: mitochondrial transcription factor A; TLR4: Toll-like receptor 4; α-syn: α-synuclein.

## Gut Microbiota-Derivates That Regulate Mitochondria in the Central Nervous System

### Gut microbiota-derived metabolites

#### Short-chain fatty acids

Bacteria in the intestines ferment sugars in the absence of oxygen to make most forms of SCFAs, including acetate, butyrate, and propionate. An essential function of SCFAs is to regulate energy production and metabolism in a hot, thus maintaining energy at a stable level. G protein-coupled receptors and histone deacetylases (HDACs) are both key signaling pathways that utilize SCFAs (He et al., 2020). The HDAC family of proteases is crucial for regulating gene expression and modifying chromosomal shape, and the natural inhibitory impact of SCFAs on HDACs is concentration-dependent (He et al., 2020). Almost every cellular and physiological process in the body is regulated by G protein-coupled receptors, the largest known family of mammalian receptors. These receptors possess seven transmembrane domains. The two most significant G protein-coupled receptors for SCFAs are GPR41 and GPR43 (Brown et al., 2003). In neurodegenerative diseases, SCFAs bind to GPR41 and GPR43 on cell membranes, thus triggering nuclear factor kappa-B and other downstream pathways, or allowing them to enter cells and block HDACs, thus controlling neurological functions (Silva et al., 2020b; Qian et al., 2022).

Researchers have recently proposed that SCFAs may influence mitochondrial activity in the brain to influence the progression of neurodegeneration. One such example is propionate, which is known to slow down MS and brain atrophy in patients by changing the shape of the mitochondria in regulatory T cells and by enhancing mitochondrial respiration (Duscha et al., 2020). Sodium butyrate has been shown to improve the pathological state of AD in a variety of ways, including increasing ATP levels, restoring mitochondrial membrane potential (MMP) of astrocytes in the toxic environments caused by A, and promoting astrocyte differentiation into the A2-neuron-protective subtype (Wang et al., 2022a). Furthermore, in a motor-neuron-like cell line used as a model for ALS, butyrate has been shown to increase mitochondrial respiratory capacity and mitochondrial network, and stimulate astrocytes to release ATP as a glial transmitter (Li et al., 2022; Karbownik et al., 2023). Butyrate and propionate promote the early neurogenic differentiation of neural stem cells by activating ROS and extracellular signal-regulated kinases 1/2 (Ribeiro et al., 2020). Lactate is the principal source of pyruvate and is transmitted to neurons through the circulatory system or other cells that serve as energy metabolism substrates (Kann, 2024). Higher lactate concentrations may cause neuroinflammation, central tiredness and cognitive impairment (Kann, 2024). Researchers have shown that microglia, which were impaired in the absence of gut microorganisms in mice, may have their mitochondrial function and shape restored by acetate; furthermore, modulation of microglial phagocytosis has been shown to slow the development of disease (Erny et al., 2021).

#### Toxins

Common pathways for LPS and other endotoxins to enter the systemic circulation include the gastrointestinal tract and the blood–brain barrier, respectively, thus accelerating the pathological process associated with aging. Elevated levels of LPS have been observed in the aged human brain and in the vicinity of neurons affected by AD (Zhao et al., 2022). Recent research reported that the disruption of mitochondrial function in the brain by intestinal microbiota-derived toxins may represent a significant process that exacerbates neurodegenerative diseases (Zhao et al., 2022). LPS has been shown to cause mitochondrial failure, including a reduced oxygen consumption rate, with significant reductions in ATP production and mitochondrial coupling efficiency in mesencephalic neurons, thus resulting in dopaminergic neuronal death (Hunter et al., 2017; Esteves et al., 2023a). Additionally, 3-nitropropionic acid is a fungal toxin that inhibits complex II (succinate dehydrogenase) and reduces ATP synthesis, resulting in the degeneration of γ-aminobutyric acid (GABA)ergic medium spiny neurons in the striatum (Upadhayay et al., 2023). β-N-methylamino-L-alanine (BMAA) is a neurotoxic compound that is produced by cyanobacteria. This compound is known to cause neurodegeneration by reducing the rate of neuronal oxygen consumption, increasing the generation of ROS, and by promoting the expression of inflammatory factors to facilitate the aggregation of pathogenic proteins (Silva et al., 2020a; Vanden Berghe, 2023).

#### Secondary bile acids

Bile acids are generated as steroids when the gut bacteria interact with a host’s metabolism. Immune cells, such as monocytes, CD4^+^ T effector cells and macrophages, express these molecules primarily by their attachment to bile acid-regulated receptors, a class of nuclear and cell membrane receptors (Fiorucci et al., 2024). The neuroprotective properties of bile acids are due to their role as an agonist for the Takeda G protein-coupled receptor-5 (Huang et al., 2022). Bile acids that have been modified by microbes in the intestines are known as secondary bile acids (Ridlon et al., 2006). Research has shown that these bile acids can reduce the symptoms of AD by increasing mitochondrial autophagy (Zhang et al., 2021b). Ursodeoxycholic acid (UDCA), lithocholic acid, and deoxycholic acid are common secondary bile acids. Recent research suggests that UDCA can reduce the neuromitochondrial damage caused by 3-nitropropionic acid, thus protecting individuals with Huntington’s disease from the loss of neurons and sensory deficits (Keene et al., 2001, 2002). By regulating mitochondrial autophagy, tauroursodeoxycholic acid (TUDCA) has shown promise in protecting neuroblastoma cells against mitochondrial dysfunction and death (Fonseca et al., 2017). In a rotenone-induced mouse model of PD, TUDCA was shown to facilitate the restoration of normal mitochondrial architecture, including the elimination of mitochondrial cristae and mitochondrial swelling (Abdelkader et al., 2016).

#### Hydrogen sulfide

Hydrogen sulfide (H_2_S) can produce a wide range of effects which can be beneficial or detrimental depending on concentration (Spalloni et al., 2023) and can even influence the CNS (Wen et al., 2024). There is evidence that H_2_S signals become dysregulated with aging, and that a post-translational change called sulfhydration/persulfidation is implicated in several physiological processes. H_2_S controls mitochondrial maintenance, thus exerting impact on the progression of neurodegenerative disease. In PD, there is a clear link between increased populations of H_2_S-producing bacteria in the colon and higher quantities of H_2_S in brain fluid (Greco et al., 2021; Nie et al., 2023; Ye et al., 2024). One potential explanation for this correlation is that cells generate an excessive amount of hydrogen peroxide, which induces mitochondria to release cytochrome c. This process, in turn, elevates the concentration of iron in the cytoplasmic pool, induces the release of ROS, and ultimately contributes to the formation of α-syn oligomers and fibrils within cells (Murros, 2022). Recent studies indicate that H_2_S acts as both a neuromodulator and neuroprotective agent, influencing the functionality of various mitochondrial components such as the electron transport chain, cytochrome c oxidase, F0–F1 ATPase, and complexes I through V, thereby helping to prevent damage to the mitochondria (Kumar and Sandhir, 2020). The oxidation of H_2_S in the mitochondria results in the production of various types of active sulfur, which can affect neuronal functionality in neurodegenerative diseases. The sulfhydrylation of proteins by these sulfur ions has been shown to result in the generation of antioxidant and anti-inflammatory effects (Zhou and Wang, 2023; Zhao et al., 2024).

#### Indole-3-propionic acid

The gut-brain axis and its therapeutic implications have been the primary focus of research involving indole-3-propionic acid (IPA), a compound that is produced by the gut microbiota via the metabolism of tryptophan (Jiang et al., 2022). The regeneration and functional recovery of sensory axons can be enhanced by IPA via immune-mediated mechanisms, specifically by upregulating the G protein-coupled receptor adenosine 5′-monophosphate (AMP)-activated protein kinase (AMPK)/silent mating type information regulation 2 homolog-1 (SIRT1) pathway (Serger et al., 2022; Yin et al., 2023). Research has also indicated that IPA may have therapeutic potential for neurodegenerative diseases such as AD and Huntington’s disease (Dragicevic et al., 2011; Rosas et al., 2015). IPA exhibits substantial antioxidant and mitochondrial protection properties and can alleviate neuronal mitochondrial dysfunction by improving mitochondrial respiration rate and membrane potential while reducing the production of ROS (Jiang et al., 2022). Thus, IPA may be closely linked to neurological diseases; however, the essential mechanism underlying the beneficial effects of IPA on neurodegenerative disorders necessitates further detailed research.

#### Urolithin A

Urolithin, an intestine metabolite produced from ellagitannin-rich foods, exhibits diverse biological activities. Urolithin A (UroA) can reduce the levels of proinflammatory enzymes (such as inducible nitric oxide synthase and cyclooxygenase-2), enhance the antioxidant potential of cells, diminish oxidative stress in neurons, while concurrently inhibiting the production of cytokines such as TNF-α, IL-6, and IL-1β (Xu et al., 2018; Kim et al., 2020a). By potentially lowering the incidence of neurodegenerative disorders in diabetic patients, UroA therapy could reduce Aβ-induced mitochondrial calcium influx and the accumulation of ROS (Lee et al., 2021). Moreover, in a rat model of PD, UroA was shown to reduce oxidative stress by enhancing mitochondrial aldehyde dehydrogenase activity, hence providing neuroprotective effects (Kujawska et al., 2019).

#### Niacinamide or nicotinamide adenine dinucleotide precursors

NAM is a prevalent metabolite of commensal microbes and is an amide form of vitamin B3 (Magnúsdóttir et al., 2015). By promoting the fission and subsequent clearance of mitochondrial autophagy, the conversion of intracellular NAM to nicotinamide adenine dinucleotide (NAD^+^) can improve mitochondrial integrity via a salvage mechanism (Jackson et al., 1995; Kang and Hwang, 2009). The NAM-producing bacterium Akkermansia muciniphila has been shown to improve both motor neuron survival and ALS symptoms by enhancing mitochondrial function and integrity (Blacher et al., 2019). Conversely, the intestinal microbiota also exhibits symbiotic metabolic reactions and are involved in the metabolism of host NAD and ultimately, can facilitate the binding of NAD^+^ precursor molecules, thereby enhancing mitochondrial function and generating protective effects against neurodegeneration (Fang et al., 2017, 2019b; Shats et al., 2020; Wang et al., 2021; Chellappa et al., 2022). Therefore, NAM and NAD^+^ precursors may represent promising therapeutic interventions for neurodegenerative disorders.

#### Gut microbiota-derived neurotransmitters and hormones

Neurotransmitters are chemical messengers that convey information between neurons and are essential for efficient functionality in the CNS and exert impact on human behavior. Neurotransmitters can be synthesized by either the CNS or peripheral organs, such as the kidneys and intestines; consequently, it is difficult to identify their precise source in blood and cerebrospinal fluid. Curiously, some types of gut bacteria can even generate neurotransmitters, and the gut microbiome can influence circuits that deal with these chemicals (Chen et al., 2022). Some bacteria, such as *Bifidobacterium spp*. or *Lactobacillus spp*., create inhibitory neurotransmitters such as gamma-aminobutyric acid (GABA). *Bacillus spp*., *Saccharomyces spp*., and *Escherichia spp*. are capable of producing noradrenaline. Other bacteria, such as *Lactobacillus spp.* produce acetylcholine. In addition, many species of bacteria, including *Streptococcus*, *Enterococcus*, *Candida*, and *Escherichia spp*., generate serotonin, while *Bacillus spp*., *Proteus vulgaris*, and *Serratia marcescens* are the main producers of dopamine (Cenit et al., 2017; Dinan and Cryan, 2017b; Strandwitz, 2018). Although the metabolism and production of some neurotransmitters are modulated by the gut microbiota, the specific mechanisms underlying the synthesis of these neurotransmitters by the gut microbiota have yet to be fully elucidated (Chen et al., 2022). Since these microbial neurotransmitters are unable to penetrate the blood–brain barrier and influence brain physiology and pathology, their impacts on the body are viewed as being more localized than peripheral. Nevertheless, the blood-brain barrier can be breached by certain neurotransmitter precursors, specifically amino acids. *Bacillus infantilum* has been shown to increase the amounts of the amino acid tryptophan in the blood; this represents the building block for serotonin. The brain can convert tryptophan into serotonin to alter brain chemistry (Boonchooduang et al., 2020). Similarly, *Bifidobacteria* can elevate the levels of phenylalanine, a precursor to the amino acid tyrosine, which acts as a precursor to two neurotransmitters in the brain: dopamine, and norepinephrine (Bull-Larsen and Mohajeri, 2019). Therefore, alterations in the abundance of bacteria that can produce precursors in the intestinal microbiota regulate the availability of monoamine neurotransmitters in the brain, thereby indirectly affecting the functional activities of the brain (Strandwitz, 2018).

Recent research has shown that some of these neurotransmitters are involved in mitochondrial function in the CNS (Chen et al., 2021). For example, high concentrations of glutamate have been shown to impair mitochondrial function in human neuroblastoma SHSY5Y cells by increasing the production of ROS and intra-mitochondrial calcium, thus promoting the opening of mitochondrial permeability transition pores, and dissipating the MMP (Dubey Tiwari et al., 2023). In addition, glutamate causes AMPK phosphorylation and subsequently promotes the uptake of glucose; this can increase mitochondrial respiration and increase intracellular levels of ROS, thus resulting in neuronal cytotoxicity (Lin et al., 2023). Elevated levels of dopamine can inhibit the activity of respiratory complex I in human neuroblastoma cells, thus resulting in mitochondrial dysfunction and depolarization within the rat striatum. This phenomenon may be linked to the development of certain neurological disorders (Brenner-Lavie et al., 2008; Czerniczyniec et al., 2010). Furthermore, GABA interferes with mitochondrial functionality, leading to reduced levels of GABAergic signaling and impairments in social behavior. This neurotransmitter has the capability to penetrate mitochondria, facilitating the synthesis of nicotinamide adenine dinucleotide phosphate (NADH) and succinate for the tricarboxylic acid cycle, whereas hyperactive mitochondria tend to sequester cellular GABA (Kanellopoulos et al., 2020). Other research has indicated that serotonin enhances mitochondrial biogenesis and stimulates the production of ATP in the cortical neurons of rodents, thus mitigating the neurotoxic impacts of oxidative stress (Fanibunda et al., 2019; Fanibunda and Vaidya, 2021). BDNF is conducive to neuroprotection against mitochondrial inhibition via the inhibition of 3-nitropropionic acid-induced autophagy; this process is dependent on the induction of p62 expression by mammalian target of rapamycin/c-Jun (Chen et al., 2017b). Ghrelin is an intestinal peptide hormone that is regulated and secreted by commensal bacteria and mediates connections among the intestinal microbiota, mitochondria and neurodegenerative diseases (Leeuwendaal et al., 2021). Ghrelin was previously shown to ameliorate Aβ-induced mitochondrial membrane depolarization, increase neuronal survival, and exert neuroprotective effects in an animal model of AD (Martins et al., 2013).

### Gut microbiota-regulated microRNAs

The importance of miRNAs that correlate with mitochondrial dysfunction, neuroinflammation and dopaminergic neuronal death with CNS integrity has been well established (Leggio et al., 2017; Choi et al., 2018). Interestingly, some studies have shown that gut microbes may produce or modulate miRNA expression in the brain to indirectly regulate mitochondrial homeostasis (Guedes et al., 2023). Animals that either germ-free or have had their microbiome depleted by antibiotics may show abnormal miRNA expression patterns in the amygdala and prefrontal cortex (Hoban et al., 2017). It is possible that microbiota in the intestines increases the levels of miR-206-3p in the brain, which in turn increases anxiety-like behavior in rats by promoting mitochondrial and synaptic degeneration in the hippocampus by downregulating Glu/Asp-rich carboxy-terminal domain 2/serine/threonine kinase (Li et al., 2023). One possible explanation is that certain genes in the brain may be targeted by interaction between the gut microbiota and miRNAs, which could potentially impact mitochondrial activity and neurodegeneration (Guedes et al., 2023).

Metabolites, neurotransmitters, hormones, and miRNAs derived from or controlled by the intestinal microbiota are crucial mediators that influence the development of neurodegenerative diseases by targeting mitochondria in the CNS. This suggests the existence of an intimate relationship between the gut microbiota and these disorders.

## Mechanistic Regulation of Mitochondrial Function by Gut Microbiota-Derivates During Neurodegeneration

The pathological relationship between gut microbiota and mitochondria influences various neurodegenerative disorders, including AD, PD and ALS. This effect could be mechanically connected to the several metabolites generated by microorganisms that can stimulate or inhibit pertinent signaling pathways or molecules in the CNS, and relate to the mitochondria (**Figure 3** and **Additional Table 1**; Li et al., 2017).

### SIRT1/PGC-1α/NRF

**Figure 4:**
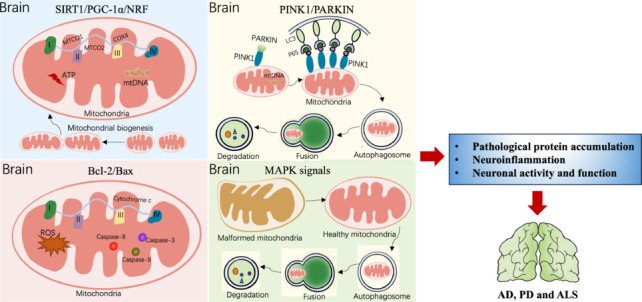
The role of SIRT1/PGC-1α/NRF, PINK1/PARKIN, Bcl-2/Bax and MAPK signaling pathways in the regulation of mitochondrial function. The signaling pathways of SIRT1/PGC-1α/NRF, PINK1/PARKIN, Bcl-2/Bax and MAPK may influence the progression of neurodegeneration. Activation of SIRT1/PGC-1α/NRF contributes to the synthesis of mitochondrial DNA (mtDNA), increases the expression of key molecules related to the mitochondrial electron transport chain including MTCO1, MTCO2 and COX4, promotes energy metabolism, and mediates mitochondrial biogenesis. PINK1/PARKIN can mediate mtDNA release and mitophagy. Bcl-2/Bax promotes the release of mitochondrial cytochrome c responsible for mitochondrial respiratory chain complex III and complex IV, enhancing the activity of caspase-8, caspase-9 and caspase-3 and the production of ROS. MAPK or its downstream signaling molecules regulate mitochondrial metabolism, mitophagy and maintain mitochondrial morphology. Created with Procreat 5.3.6 and Microsoft PowerPoint. AD: Alzheimer’s disease; ALS: amyotrophic lateral sclerosis; ATP: adenosine triphosphate; Bcl-2/Bax: B-cell lymphoma-2/Bcl-2-associated X protein; CoX4: cytochrome c oxidase subunit 4; LC3: light chain 3; MAPK: mitogen-activated protein kinase; mtDNA: mitochondrial DNA; MTCO1: mitochondrially encoded cytochrome c oxidase l; PD: Parkinson’s disease; PINK1/PARKIN:PTEN-induced putative kinase1/parkin; ROS: reactive oxygen species; SIRT1/PGC-1α/NRF: silent mating type information regulation 2 homolog-1/peroxisome proliferator-activated receptor γ coactivator 1 alpha/nuclear factor erythroid 2-related factor.

The activity of peroxisome proliferator-activated receptor γ coactivator 1 alpha (PGC-1α) is known to control the establishment of mitochondria. Following activation by either phosphorylation or deacetylation, PGC-1α initiates the activation of nuclear factor-erythroid 2-related factor 1 (NRF1) and NRF2, respectively, followed by mitochondrial transcription factor A (TFAM). Activation of the PGC-1α/NRF/TFAM pathway leads to the production of new mitochondria, mtDNA and proteins (Li et al., 2017). SIRT1 is predominantly expressed in the nucleus. When activated, SIRT1 encourages the deacetylation of PGC-1α, thus facilitating mitochondrial biogenesis and respiration (Tang, 2016). Neurodegenerative diseases have been found to be closely associated with the dysregulation of PGC-1α (Gerhart-Hines et al., 2007). Currently, available data suggests that substances generated by the gut microbiota can regulate the SIRT1/PGC-1α/NRF signaling pathway in mitochondria, a mechanism that has been implicated in the development of neurodegenerative diseases (St-Pierre et al., 2006) (**Figure 4**).

Butyrate treatment has been shown to activate the PGC-1α signaling axis in a cell model of ALS, thus resulting in the upregulation of the key molecules in the mitochondrial electron transport chain (cytochrome c oxidase subunits 1 and 2, and cytochrome c oxidase subunit 4). As a result, the mitochondria produce more bioenergy and the progression of ALS in mice is slowed down (Li et al., 2022). Furthermore, research has demonstrated that the ability of the biogenesis of astrocytes can be enhanced by butyrate, and neuroprogenitors, such as PC12-NeuroD6 cells and A embryonic hippocampal progenitor cells. This was achieved by elevating the expression level of proteins in the PGC-1α/NRF1/TFAM signaling pathway, which can either improve the cognitive impairment caused by AD or enhance the neuronal differentiation competency of these cells (Uittenbogaard et al., 2018; Wang et al., 2022a). Serotonin, a hormone that is known to regulate mitochondrial biogenesis and cortical neuron functionality upstream, exerts a neuroprotective impact by increasing mitochondrial respiratory capacity, oxidative phosphorylation efficiency, and mtDNA via the SIRT1/PGC-1α axis (Fanibunda et al., 2019). NAM and Akkermansia muciniphila can modulate mitochondrial biogenesis, oxidative stress, and electron transport chain activity owing to the significant proportion of common binding sited for PGC-1α in their response-related gene promoters and an increase in SIRT activity, thereby slowing the progression of ALS and PD (Virbasius et al., 1993; Jia et al., 2008; Blacher et al., 2019). In addition, AMPK is indirectly stimulated or PGC-1α deacetylation occurs as the result of the NAM precursor NA riboside activating SIRT1 via an increase in NAD levels. Mitochondrial biogenesis can be accelerated via the activation of the SIRT1/AMPK/PGC-1α axis (Bergeron et al., 2001; Uittenbogaard and Chiaramello, 2014). In addition to protecting animal models of PD and enhancing autophagy flux, H_2_S has been shown to enhance SIRT1 activity and promote PGC-1α deacetylation via H_2_S-mediated sulfhydryl hydration (Li et al., 2020). Research involving a model of PD demonstrated that UroA protected dopaminergic neurons by inducing mitochondrial autophagy via the SIRT1/PGC-1α signaling pathway (Liu et al., 2022). Moreover, research involving several models of PD demonstrated that TUDCA, an encouraging drug that partially inhibits ROS-mediated damage, was able to regulate mitochondrial biogenesis by altering the NRF-2 signaling pathway (Moreira et al., 2017).

### PINK1/PARKIN

PINK1 and PARKIN proteins regulate the targeted clearance of defective or surplus mitochondria and facilitate the secretion of mtDNA to enhance neuroprotective and anti-inflammatory characteristics in neurodegenerative disorders. Selective autophagy, also known as mitochondrial autophagy, is driven by PINK1 and regulates the translocation of PARKIN in injured mitochondria. As humans age, the muscles and brain undergo increased levels of mitochondrial autophagy. However, this process is rendered useless when the PINK1/PARKIN signaling pathway is disrupted; this process can impede the mechanism responsible for this vital quality check and permits the build-up of damaged mitochondria (Cornelissen et al., 2018). Growing evidence indicates that the impairment of mitochondrial autophagy is a significant component of various human neurodegenerative disorders, especially PD (Quinn et al., 2020).

Certain metabolites produced from the gut microbiota have been demonstrated to modulate the PINK1/PARKIN signaling pathway, thus influencing neurodegenerative diseases. TUDCA may enhance the elimination of dysfunctional mitochondria by stimulating the PINK1/PARKIN or AMPK/mammalian/mechanistic target of rapamycin (mTOR) pathways to avert neurodegeneration (Fonseca et al., 2017; Rosa et al., 2017; Qi et al., 2021). Moreover, PARKIN can undergo physiological modification by H_2_S, a process known as sulfonation, to enhance its catalytic activity. The expression of H_2_S in the brains of individuals with PD was previously shown to be significantly diminished, thus indicating that this reduction may be harmful. Consequently, H_2_S is acknowledged as a prospective treatment for neurodegenerative disorders (Vandiver et al., 2013). Moreover, probiotics have been shown to enhance mitochondrial autophagy via the PINK1/PARKIN-dependent pathway, thus inhibiting the course of PD, including the enhancement of motor function and lifespan (**Figure 4**; Hawrysh et al., 2023).

### B-cell lymphoma-2/Bcl-2-associated X protein

Owing to aerobic metabolism, the CNS is susceptible to oxidative stress during brain aging (Tabassum et al., 2020). Mitochondria are essential in cellular apoptosis when induced by oxidative stress by activating proapoptotic proteins and inhibiting antiapoptotic proteins, thus resulting in neuronal cell death. Bcl-2-associated X protein (Bax) and Bak, located on the mitochondrial outer membrane, exert proapoptotic activity mediated by members of the family of B-cell lymphoma-2 (Bcl-2) proteins. This action induces cellular apoptosis and regulates mitochondrial apoptosis. The transcription of mitochondrial Bax and Bak is reversed in the cytoplasm by antiapoptotic Bcl-2 proteins, thus ensuring cell survival. In addition, mitochondrial cytochrome c protein is released due to Bax/Bak-dependent permeabilization of the outer mitochondrial membrane, facilitating the activation of caspase-9. Neurodegenerative illnesses are associated with the development of apoptotic bodies, plasma membrane blebbing and cellular atrophy, all of which are regulated by the caspase cascade (**Figure 4**; Tatton et al., 2003; Wolf et al., 2022).

There have been reports of compounds derived from the gut microbiota that can target Bcl-2/Bax signaling and influence the course of neurological diseases. For example, it has been demonstrated that H_2_S activates antioxidant enzymes, which in turn restrict reactions including free radicals. This antioxidant effect, which is beneficial for neuroprotection, is mediated by genes associated with apoptosis, including p53 and Bcl-2/Bax (Tabassum et al., 2020). Studies have indicated that neurotoxicity is caused by the release of cytochrome c by mitochondria. In the mitochondrial respiratory chain cytochrome c controls the transfer of electrons from complex III to complex IV (Murros, 2022). In PD, the production of ROS encourages the development of α-syn oligomers and fibrils while cytochrome c exhibits significant peroxidase activity (Belikova et al., 2006; Bayir et al., 2009; Kumar et al., 2016). In addition, UDCA reduces the activities of caspase-8, caspase-9 and caspase-3 while altering the expression of Bax and Bcl-2 to prevent rotenone-induced apoptosis in models of PD. It is also possible that increased levels of ghrelin can slow or stop the progression of neuronal loss in PD (Rees et al., 2023) by re-establishing mitochondrial function by regulating Bcl-2/Bax to limit the activation of caspase-3 (Jiang et al., 2008; Dong et al., 2009). Ghrelin may represent a potential new treatment option for neurodegenerative diseases due to its anti-inflammatory and antiapoptotic actions associated with mitochondrial regulation.

### Mitogen-activated protein kinase signaling pathway

The mitogen-activated protein kinase (MAPK) pathway plays a pivotal role in the progression of neurodegenerative disorders. It is known that several members of the MAPK family, including c-Jun NH2-terminal kinase (JNK), p38, and extracellular signal-regulated protein kinase (ERK), can exert impact on the transcriptional control of several genes encoding proinflammatory cytokines. Moreover, MAPK and its downstream signaling molecules can influence cell survival or death by regulating mitochondrial metabolism and the induction of mitophagy (Javadov et al., 2014; Grassi et al., 2019). Mitochondrial morphological abnormalities and dysfunction in mouse models of PD have been reported to be associated with mitophagy and impaired MAPK/ERK signaling (Liu et al., 2021). In the advent of mitochondrial stress, the levels of intracellular ROS increases; this causes cellular oxidative stress, and activates the JNK, ERK and p38 MAPK pathways to eventually induce cellular apoptosis (**Figure 4**; Ki et al., 2013; Cao et al., 2021).

The activity of MAPK family members (JNK, p38 and ERK) in the hippocampus and cortex was shown to be reduced following the systematic chronic treatment of severe models of AD with H_2_S. This reduction in activity triggered anti-inflammatory responses and reduced the size of Aβ plaques. Consequently, cognitive impairment was ameliorated (Vandini et al., 2019). Furthermore, researchers have shown that H_2_S provides a protective effect on microglia against Aβ-induced toxicity in AD by enhancing cell proliferation, reducing inflammation and maintaining mitochondrial functionality through the p38 and JNK-MAPK pathways (Liu and Bian, 2010). In a rotenone-induced model of PD, H_2_S suppressed cell injury in a concentration-dependent manner by regulating mitochondrial ATP-sensitive potassium, thereby inhibiting the P38-JNK-MAPK phosphorylation pathway (Hu et al., 2009).

### Other pathways and molecules

The relationship between microbiota and mitochondria can also modulates other signaling pathways or chemicals, thus influencing neuropathologies. The mitochondrial melatonergic pathway is considered fundamental to cellular function; blocking this pathway elevates ROS levels, which in turn induces miRNA expression to modify the gene expression profile; this process is intimately associated with the pathogenesis of ALS. Recent data has demonstrated that the gut bacteria, particularly butyrate derivatives, might enhance the activity of systemic and central mitochondrial melatonergic pathways, thus facilitating numerous advantageous effects for neurodegeneration (Anderson, 2022). Butyrate was previously shown to alleviate the symptoms of PD via its ability to inhibit HDAC and directly influence mitochondrial function, predominantly by mitigating the detrimental effects of ceramides, a putative contributor to the pathophysiology of PD (Plotegher et al., 2019; Bjørklund et al., 2020). Furthermore, bile acid derivatives can repair mitochondrial damage by regulating the activation of nuclear factor-erythroid 2-related factor 2 and p62/LC3B-mediated autophagy to mitigate the symptoms of PD (Huang et al., 2022). TUDCA, a powerful antiapoptotic agent and mitochondrial stabilizer, has been demonstrated to reduce the production of ROS *in vivo*, thus providing a protective effect against neurotoxicity by inhibiting the expression of Hsp27, a heat shock gene implicated in the activation of ROS in the midbrain and striatum, thus enhancing the antioxidant capacity of the internal environment (Rosa et al., 2018). H_2_S can safeguard animal models of AD from cognitive decline by reducing the activity γ-secretase by diminishing the accumulation of Aβ in mitochondria (Zhao et al., 2016b). Researchers have found that UroA triggers an overload of mitochondrial Ca^2+^ and an increase in mtROS, ultimately reducing the production of Aβ in AD by lowering the expression levels of amyloid precursor protein and its processing enzyme, β-secretase 1. Furthermore, UroA inhibits the transcription of transglutaminase type 2, thereby disrupting the interactions between mitochondria and the endoplasmic reticulum (Garcia-Alloza et al., 2021; Lee et al., 2021).

In summary, interplay between the gut microbiota and mitochondria can exert significant influence on progression and outcome of neurodegenerative diseases by impacting mitochondrial function to regulate neuroinflammation, while also establishing a complex signaling network involving multiple interacting pathways. Nonetheless, the precise methods by which these signaling molecules mediate interactions between the gut microbiota and mitochondria in the CNS require additional clarification.

## Microbiota‐Mitochondria Crosstalk Modulates the Innate Immune Response to Influence Neurodegeneration

**Figure 5:**
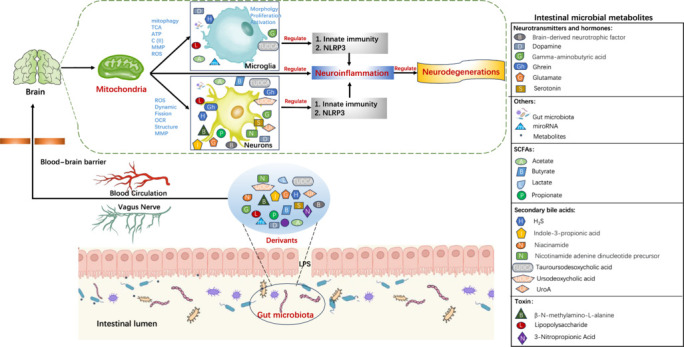
Modulation of the innate immunity by gut microbiota–mitochondria crosstalk. Gut microbiota-derived metabolites cross the blood–brain barrier and enter the brain via the blood circulation and vagus nerve to target and regulate the functions of mitochondria in the CNS. There, metabolites affect the activation of the innate immune system and the NLRP3 complex in microglia and neurons to regulate neurodegeneration. Created with Procreat 5.3.6 and Microsoft PowerPoint. ATP: Adenosine triphosphate; C(II): cytochrome c oxidase subunit ll; ETC: electron transport chain; LPS: lipopolysaccharide; MMP: mitochondrial membrane potential; NLRP3: NLR family, pyrin domain containing protein 3; OCR: oxygen consumption rate; ROS: reactive oxygen species; TCA: tricarboxylic acid cycle.

The innate immune response in the CNS is a critical factor that contributes to neuroinflammation, a hallmark of neurodegenerative disorders (Leng and Edison, 2021; Calma et al., 2024). The promotion of innate immunity by dysfunctional mitochondria under conditions of neurodegeneration has been described previously. Over recent years, researchers have reported that the oxidative/inflammatory damage caused by disorders of the gut microbiota and its metabolites frequently results in impaired functionality of the gut barrier and the blood–brain barrier. This allows inflammation to “escape” and ultimately disrupt mitochondrial function in the brain (Roy Sarkar and Banerjee, 2019; Silva et al., 2022). The ability of the gut microbiota to regulate innate immunity within the CNS is largely facilitated by mitochondria (Song and Fan, 2023). The development of neurodegenerative disease is influenced by microbiota–mitochondria crosstalk, which controls the innate immune response through effects on cellular activity in the CNS (**Figure 5**).

### Microbiota‒mitochondria crosstalk regulates microglial function

In both healthy and diseased brains, the microglia, the densest population of innate immune cells in the CNS, are involved in a wide variety of processes. The regulation of diseases such as neuroinflammation and the maintenance of a healthy tissue microenvironment are both facilitated by microglia. Microglia play a key role in pathological neuroinflammation during neurodegeneration; gut microbe-mitochondria crosstalk may regulate the function and development of microglia (Erny et al., 2015; Colonna and Butovsky, 2017; Prinz et al., 2019; Guedes et al., 2023).

Microglia proinflammatory reactions are mediated by the mitochondrial targeting of certain compounds from the gut microbiota. A lifelong lack of host microbes has the potential to alter epigenetic imprinting in the microglia by influencing histone acetylation and the expression of methylation markers (H3K4me3 and H3K9ac) at specific promoter regions of metabolism-related genes; these alterations are linked to significant alterations in mitochondrial function. Reduced levels of ATP and impaired mitochondrial-specific respiratory chain complex II levels are the results of a major impact on microglia mitochondrial metabolic networks, which include components of the tricarboxylic acid cycle and purine metabolism. Mitochondrial mass, membrane potential and ROS levels are all affected by these biological changes in the microglia, which in turn affect the shape, proliferation, and activation of microglia (Erny et al., 2021). In order to enhance the inflammatory response, activated microglia have the ability to detect and remove clumped pathogenic proteins such as tau, Aβ, and α-syn (Leuner et al., 2012; Devos et al., 2013). Activated microglia exacerbate the severity of motor symptoms and the progression of neurological disorders by releasing the NLRP3 inflammasome, leading to the generation of ROS and the secretion of proinflammatory cytokines (González and Pacheco, 2014; Fan et al., 2020). SCFAs generated from the microbiome also affect microglial metabolic pathways and functionality under both healthy and disease conditions. Acetate enhances microglial growth and immune activity by regulating mitochondrial function. Research has revealed that acetate-dependent adaptation addressed metabolic deficits in microglia from germ-free animals in a stable state and influenced the progression of dementia by inhibiting microglial phagocytosis (Erny et al., 2021). The synthesis of acetate has been shown to diminish the phagocytosis of the Aβ peptide in mouse models of AD, resulting in the enhanced accumulation of Aβ, which in turn impaired mitochondrial maintenance and elevated the generation of ROS (Pinho et al., 2014). Impaired mitochondria and ROS generation subsequently exacerbated oxidative stress and neuroinflammation.

Conversely, some products from the gut microbiota can mitigate the innate immunity activated by microglia. H_2_S has been demonstrated to inhibit Aβ-induced toxicity in microglial cells and to maintain mitochondrial activity, leading to reduced inflammation and enhanced cell proliferation (Liu and Bian, 2010). A study utilizing an MPTP-induced model of PD revealed that promoting the selective degradation of dysfunctional mitochondria, termed mitochondrial autophagy, suppressed activation of the NLRP3 inflammasome in microglia, preserved dopaminergic neurons, and enhanced behavioral outcomes (Ahmed et al., 2021). UroA was previously identified as a promoter of mitochondrial autophagy, mitigating the proinflammatory response in BV2 microglia and diminishing activation of the NLRP3 inflammasome in both LPS-induced BV2 microglia and MPTP-induced models of PD. Pharmacological disruption of the mitochondrial autophagy component of microglia diminished the neuroprotective efficacy of UroA in a mouse model of PD (Qiu et al., 2022). In a model of AD, UroA was demonstrated to function as an inducer of mitochondrial autophagy, enhancing cognitive function by promoting microglial plaque clearance, suppressing neuroinflammation, and removing hyperphosphorylated tau (Fang et al., 2019a). The anti-inflammatory impact of mitochondrial autophagy may connect mechanistically to the PINK1/PARKIN signaling pathway. The absence of PINK1 or PARKIN contributes to neuroinflammation by initiating an inflammatory response and enhancing the survival of activated microglia (Wu et al., 2021). TUDCA therapy, which elevates the levels of antioxidant enzymes and PARKIN, was demonstrated to reduce glial cell activation and mitigate the detrimental effects of MPTP by downregulating neuroinflammation and enhancing annexin-A1 expression (Dionísio et al., 2015; Mendes et al., 2019).

### Microbiota‒mitochondria crosstalk regulates neuronal function

Although neurons are not immune cells, they play an important role in controlling innate immunity in the CNS. Neurons contribute to the production of inflammatory cytokines via their expression of pattern recognition receptors, such as TLR4 and TLR3. Furthermore, they also trigger the NLRP3 inflammasome, thereby initiating innate immunity in a manner that is independent of microglial involvement (Fann et al., 2018). Two major pathophysiological hallmarks of neurodegenerative diseases, are neuronal mitochondrial failure and neuroinflammation, are known to interact with one another. An increasing body of research now indicates that the communication between gut bacteria and mitochondria can lead to neuroinflammation by influencing the abnormal progression of neuronal damage and the regulation of the inflammatory response.

Emerging evidence has revealed that toxins derived from the intestinal microbiota can impair neuronal mitochondria, thus aggravating neuroinflammation. The exposure of mesencephalic neurons to *Alphaproteobacterium* was shown to increase the expression and aggregation of α-syn, thus resulting in dysregulated mitochondrial dynamics; this generated a positive feedback loop of innate immune signals in neurons (Magalhães et al., 2023). Previous research reported that the expression of LPS in the colon and plasma of PD patients was elevated, thus leading to activation of the inflammatory response (Perez-Pardo et al., 2019). One possible link between this activation and the capacity of LPS to enhance mitochondrial ROS production and hinder mitochondrial fission is that it could trigger the release of IL-1β, a known inflammatory agent. Furthermore, inflammation of the innate immune system and the oligomerization of α-syn in PD are mediated by mitochondrial dysfunction caused by LPS. Crucial regulators of the formation and activation of the NLRP3 inflammasome include the aggregation of pathological protein, neurotoxins, and mitochondrial dysfunction. This leads to the release of IL-18 and IL-1β from neurons and their pyroptotic death (Haque et al., 2020). It is important to note that the ability of primary mesencephalic neurons to activate neuronal innate immunity in response to LPS is conditional on their mitochondria acting in a functional manner. Previously, researchers found that LPS could not activate innate immunity or accumulate α-syn in cell lines that were devoid of functional mitochondria. Thus, in LPS-induced PD, mitochondria provide the groundwork for activation of the innate immune system (Esteves et al., 2023a). BMAA, another toxin, may trigger the neuronal innate immune response via two pathways during the progression of AD and PD. BMAA can directly induce mitochondrial dysfunction in neurons and activate TLRs, thus leading to the increased release of inflammatory proteins, including mature IL-1β, and the NLRP3 inflammasome via mtDAMPs. Furthermore, BMAA might indirectly impair mitochondrial function in neurons by increasing the aggregation of pathogenic proteins, thus resulting in the activation of innate immunity in the CNS (Silva et al., 2020a; Esteves et al., 2023b). Vanden Berghe (2023) postulated a cohesive mechanism for AD and PD in which BMAA, a microbial toxin, induces mitochondrial malfunction and activates neuronal innate immunity, ultimately resulting in disorders related to tau, Aβ and α-synuclein disorders.

Certain compounds produced by gut microorganisms can exhibit anti-inflammatory properties on neurons. H_2_S demonstrated its protective role for neuronal cells against neurotoxicity caused by homocysteine (a strong risk of AD) or formaldehyde, by maintaining MMP and reducing the levels of intracellular ROS accumulation (Tang et al., 2010, 2012). A previous study used the Morris water maze test and found that mitochondria-targeted H_2_S exhibited neuroprotective effects on APP/PS1 neurons in a dose-dependent manner by enhancing energy production, improving cell viability, safeguarding mtDNA and reducing the build-up of ROS. Consequently, this action led to a reduction in Aβ deposition within the brain and enhanced memory function (Zhao et al., 2016a). To summarize, during the progression of neurodegenerative illnesses, interaction between the gut microbiota and mitochondria is critical for regulating innate immune responses in the CNS, which in turn impacts neuroinflammation. While previous research has investigated the impact of microbiota–mitochondria interaction on neuroinflammation in a general manner, very few of these studies have focused on particular brain regions or other cell types.

## Potential Strategies for Neurodegeneration: Targeting Microbiota–Mitochondria Crosstalk

**Figure 6:**
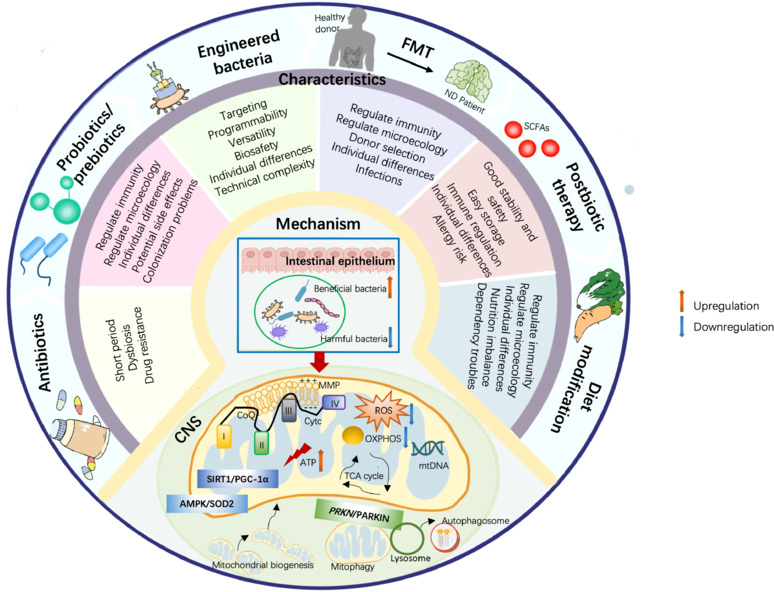
The characteristics and mechanism of existing potential strategies for neurodegeneration by targeting microbiota-mitochondria crosstalk. The existing potential strategies include the utilities of antibiotics, probiotics and prebiotics, engineered bacteria, FMT, postbiotic therapy and dietary modification. These strategies affect MMP, the production of OXPHOS, ROS and ATP, mtDNA synthesis, TCA cycle, mitochondrial biogenesis, and mitochondrial electron transport chain, and regulate the signaling pathways of SIRT1/PGC-1α, AMPK/SOD2 and PRKN/PARKIN by improving gut microbiota to alleviate the symptoms of neurodegeneration. Created with Procreat 5.3.6 and Microsoft PowerPoint. ATP: Adenosine triphosphate; AMPK/SOD2: adenosine 5′-monophosphate (AMP)-activated protein kinase/superoxide dismutase 2; CNS: central nervous system; CoQ: coenzyme Q10; Cyt c: cytochrome c; FMT: fecal microbiota transplantation; MMP: mitochondrial membrane potential; mtDNA: mitochondrial DNA; OXPHOS: oxidative phosphorylation; PRKN/PARKIN: parkin RBR E3 ubiquitin protein ligase gene; ROS: reactive oxygen species; SCFAs: short-chain fatty acids; SIRT1/PGC-1α: silent mating type information regulation 2 homolog-1/peroxisome proliferator-activated receptor γ coactivator 1 alpha; TCA: tricarboxylic acid cycle.

By acting through the gut–brain axis, the gut microbiota and its metabolites play a significant role in aging and neurodegenerative disorders. Little is known about how the gut microbiota influences brain and circulatory systemic metabolism, new evidence suggests that mitochondria act as the primary mediators of this bacterial role in cerebral activity. One possible treatment approach for neurodegenerative diseases could be to modify the gut microbiome in order to target microbiota–mitochondria crosstalk. This could be achieved by dietary changes, exercise, environmental pollutants, stress and other genetic and environmental variables (**Figure 6** and **Additional Table 2**). Therapeutic strategies that targeting microbes can generally be divided into three approaches. The first approach involves the elimination of pathogenic microbes via antibiotic treatment. The second approach treats microbes and their products as therapeutic agents to supplement patients. This can be achieved by supplementing with probiotics and prebiotics, using engineered microbes, administering small molecules derived from microbes, such as bacterial metabolites, or by transplanting microbiota from healthy subjects to patients. The general goal is to establish a stable and coordinated microbial community to replace an undesirable microbial composition, thereby restoring the functionality of a healthy gut microbiome (Ratiner et al., 2024). The third approach is to indirectly regulate microbial homeostasis by adjusting dietary structure or by administering drugs that can influence the microbiota.

**Additional Table 2:** Summary of treatment approaches for neurodegenerative diseases that target the microbiota-mitochondria crosstalk

Treatment approach			Gut microbiota effect	Mitochondrial effect	Neuronal effect	Neurodegenerative disease	Reference
Antibiotics	Tetracycline	Minocycline	Altered the gut microbiota	Increased mitochondrial quadruple complex activities and antioxidant capacity; Augmented mitochondrial membrane potential and ATP level	Neuroprotection; Limited inflammation and oxidative stimulation	hd, pd	Budni et al., 2016; Motaghinejad et al., 2023
		Doxycycline		Prevented mitochondrial membrane depolarization and intracellular oxidative stress	Neuronal protection; Reduced neuroinflammation and oxidative stress; Microglial activation	PD	Paldino et al., 2020; Dominguez-Meijide et al., 2021
	Rapamycin			Promoted autophagy; Alleviated mitochondrial damage	Diminished neuronal loss; Improved motor system function; Augmented motor neuron degeneration	PD	Zhang et al., 2011; Pupyshev et al., 2019; Gonzalez-Alcocer et al., 2022
	Rifampicin			Alleviated mitochondrial damage; Decreased mitochondria ros	Reduced α-synuclein aggregation; Anti-neuroinflammation; Suppressed neuronal apoptosis	PD	Liang et al., 2017; Reglodi et al., 2017; Socias et al., 2018
	Chloramphenicol			Regulated mitochondrial complex I; Attenuated ROS production	Increased neuronal activity; Inhibited neurotoxins	PD	Han et al., 2019
	Niclosamide			Promoted mitochondrial depolarization	Neuroprotection	PD	Barini et al., 2018
Probiotics	Acidophilus probiotic		Repaired the gut microbiota and maintained immune homeostasis	Alleviated mitochondrial dysfunction	Alleviated learning and memory deficits	AD	Beltagy et al., 2021
	Lactobacillus salivarius			Restored mitochondria function; Inhibited ROS	Improved motor function; Neuroprotection	PD	Nurrahma et al., 2021
	Bifidobacterium			Decreased mitochondrial ROS	Anti-neuroinflammation	PD	Magistrelli et al., 2019
	Saccharomyces boulardii and Lactococcus lactis Lacticaseibacillus rhamnosus			Facilitated mitochondrial autophagy Regulated mitochondrial oxidation	Alleviated impaired movement Stabilized energy metabolism; Improved lipid homeostasis	PD ALS	Hawrysh et al., 2023 Labarre et al., 2022
Prebiotics	Inulin		Excited the growth or activity of specific gut bacteria	Reduced ROS; Improved cytochrome c oxidoreductase and ATPase activities	Alleviated oxidative damage; Resisted neurotoxicity	PD	Krishna and Muralidhara, 2015, 2018
Fecal microbiota transplantation			Re-established a healthy gut microbiota community	Reprogrammed mitochondrial metabolism; Ameliorated mitochondria impairments	Alleviated neuronal and synaptic loss; Inhibited cognitive impairment; Repaired blood-brain barrier integrity	ad, PD	Sun et al., 2018; Fujii et al., 2019; Zhao et al., 2021b
Diet modification	A low-carb, high-fat dietary regimen		Maintained a beneficial gut microbiota composition	Regulated mitochondrial biogenesis	Energy source for brain cells; Neuroprotection	ad, PD	Augustin et al., 2018; Jiang et al., 2023
	A low-protein, high–carbohydrate diet Polyphenols			Unknown Regulated mitochondrial function; Ameliorated mitochondrial dysfunction	Neuroprotection Increased health and lifespan; Suppressed oxidative damage	PD	Chu et al., 2023 Dilberger et al., 2019; Rivas et al., 2022
Antimicrobial peptides			Distinguished bacteria from host cells	Unknown	Neuroprotection; Prevented amyloid protein aggregation; Ameliorated motor dysfunction	ad, PD	Kim et al., 2015; Zhang et al., 2021a
Mangosteen pericarp			Remodeled fecal microbiota composition	Restored mitochondrial function	Alleviated motor deficits	PD	Nurrahma et al., 2022
Acarbose			Restructured the gut microbiota and altered short-chain fatty acid production	Improved mitochondrial function	Slowed the progression of mitochondrial disease	ALS	Hoffmann and Spengler, 1997; Bitto et al., 2023

AD: Alzheimer's disease; ALS: amyotrophic lateral sclerosis; ATP: adenosine 5'-triphosphate; HD: Huntington's disease; PD: Parkinson's disease; ROS: reactive oxygen species.

### Antibiotics

Several antibiotics have demonstrated potential as therapies for various neurodegenerative disorders. These antibiotics exhibit a broad spectrum of activity, encompassing modulation of the gut microbiome, anti-inflammatory properties, and effects on mitochondrial enhancers. Numerous investigations have found that the tetracycline (TC) class of antibiotics can be efficacious against neurodegenerative diseases. For PD, minocycline has been investigated as a potential treatment approach. For instance, minocycline was found to enhance activities of the mitochondrial quadruple complex, total antioxidant capacity, and the levels of both MMP and ATP to limit inflammation and oxidative stimulation, thus acting as a neuroprotective agent for neurodegeneration and cognitive impairment (Budni et al., 2016; Bortolanza et al., 2018; Dominguez-Meijide et al., 2021; Motaghinejad et al., 2023). Tourville et al. (2023) recently reported that doxycycline (DOX) and demeclocycline (DMC), both TC class antibiotics, provided sustained protection for dopaminergic neurons in the mouse midbrain. These authors reported that chlortetracycline and non-tetracycline class antibiotics did not exert this effect. It is noteworthy that non-antibiotic derivatives of DOX and DMC, namely DDOX and DDMC, provided significant protection for dopaminergic neurons even in the latter stages of neurodegenerative diseases. The protective effect of TC predominantly stems from its capacity to avert intracellular oxidative stress and mitochondrial membrane depolarization. Furthermore, DOX has been shown to inhibit the aggregation of α-syn (Dominguez-Meijide et al., 2021), thus disrupting mitochondrial autophagy, leading to the buildup of defective mitochondria (Lindström et al., 2017), thus reducing the production of ROS.

Rapamycin, a novel and potent immunosuppressant, enhances autophagy by inhibiting mTOR, thereby reducing neuronal loss and enhancing the motor system (Pupyshev et al., 2019). Furthermore, co-treatment with rapamycin and trehalose was shown to exert additive effects on the promotion of autophagy *in vitro*; consistent results have also been detected *in vivo* in the substantia nigra (Gonzalez-Alcocer et al., 2022). However, the opposite effects were reported for rapamycin treatment, which augmented the degeneration of motor neurons, induced apoptosis, and caused mitochondrial damage (Zhang et al., 2011). Rifampicin demonstrated efficacy in the reduction of α-syn aggregation, exerting anti-neuroinflammatory effects, and inhibiting neuronal apoptosis by suppressing activity of the NLRP3 inflammasome in PD (Reglodi et al., 2017; Socias et al., 2018). The mechanism of action may involve the induction of robust autophagy, which is conducive to increasing the number of lysosomes, restoring mitochondrial damage and by inhibiting the production of ROS (Liang et al., 2017). The administration of chloramphenicol was found to effectively inhibit neurotoxins, such as MPTP and paraquat-induced PD pathology, and to significantly increase the activity of primary dopaminergic neurons from rat embryos. The protective effect of chloramphenicol on neurons was mediated by targeting mitochondrial complex I and by the attenuation of ROS production; paraquat-induced dopaminergic neuron loss was associated with oxidative stress and ROS via regulating mitochondrial complex I (Han et al., 2019). Moreover, the use of niclosamide, a salicylamide drug that promotes the activation of PINK1 by driving mitochondrial depolarization, also represents a potential therapeutic approach (Barini et al., 2018).

Despite the fact that antibiotic administration has been demonstrated to have an impact on neurodegenerative diseases, antibiotic therapy limited by its potential to induce antibiotic resistance. Moreover, the broad-spectrum characteristics of antibiotics may lead to the indiscriminate eradication of both helpful and pathogenic gut bacteria, thereby causing an imbalance in the microbial population and other consequences, such as irritable bowel syndrome (Mamieva et al., 2022). Consequently, it is imperative to meticulously evaluate the potential dangers of this strategy alongside an individual patient’s particular situation before administering antibiotics for the treatment of neurodegenerative illnesses in clinical practice.

### Probiotics and prebiotics

Specific intestinal microorganisms, known as probiotics, have a beneficial effect on host health by helping to restore the gut microbiome and maintain immune balance. On the contrary, prebiotics are dietary fibers that dissolve in water, promoting health in the host by stimulating the growth or activity of specific intestinal microbes. Multiple studies suggest that probiotics and prebiotics primarily influence and regulate mitochondrial function within the CNS, although the exact mechanisms by which they impact neurodegenerative diseases remain unclear (Hsia et al., 2012; Chen et al., 2017a; Mularczyk et al., 2021).

*Lactobacillus acidophilus* has the ability to enhance MMP, thus helping to alleviate mitochondrial dysfunction and reduce the cognitive impairments associated with learning and memory in AD (Beltagy et al., 2021). In a rat model of PD, probiotic supplements (a subspecies of *Lactobacillus salivarius*) have been shown to restore energy metabolism by safeguarding mitochondria from damage associated with ROS. This results in a neuroprotective effect on dopaminergic neurons and enhances motor function (Nurrahma et al., 2021). Fatty acids from *Lactobacillus rhamnosus* can penetrate mitochondria without reliance on the impaired carnitine shuttle, thus exerting influence on the mitochondrial β-oxidation mechanism. This stabilizes energy metabolism and enhances lipid homeostasis in models of ALS (Labarre et al., 2022). Furthermore, probiotics such as *Saccharomyces boulardii* and *Lactococcus lactis* can influence the mitochondrial parkin RBR E3 ubiquitin protein ligase gene (*PRKN*)/PARKIN pathway, thus enhancing the autophagy of dysfunctional mitochondria and mitigating the symptoms of patients with PD (Hawrysh et al., 2023).

Inulin supplements, as prebiotics, were shown to mitigate neurotoxicity by modulating the gut microbiota and by restoring mitochondrial function. Inulin supplementation may mitigate acrylamide-induced oxidative damage and neurotoxicity in the brains of maternal and fetal rats, thereby providing a substantial protective effect on mitochondria (Krishna and Muralidhara, 2015). In a model of rotenone-induced neurotoxicity, the administration of inulin increased the abundance of cecal bacteria in maternal mice, reduced ROS levels in maternal brain regions and inhibited rotenone-induced declines in NADH cytochrome c oxidoreductase and ATP activities (Krishna and Muralidhara, 2018). Moreover, various prebiotics have been demonstrated to indirectly influence mitochondrial function by impacting microbiota-derived SCFAs, including butyrate and propionate (Srikantha and Mohajeri, 2019). Researchers have proposed synthetic formulations that combine prebiotics and probiotics to influence the microbial-gut-brain axis for the prevention of neurodegeneration. During the onset and progression of AD, this artificial therapy increases lifespan by enhancing mitochondrial performance and reducing the oxidative stress and inflammation associated with aging, while simultaneously offering positive effects on PPAR dependence (Jalali et al., 2021).

Nonetheless, numerous issues persist regarding the application of probiotics and probiotic therapy. The safety of probiotics and prebiotics requires comprehensive evaluation. Individuals with compromised immune systems, the elderly, critically ill patients or children, may face an elevated risk of infection or complications from the use of these substances (Merenstein et al., 2023). Furthermore, interactions between probiotics or prebiotics and other pharmaceuticals could influence the efficacy of treatment (Dikeocha et al., 2022). Therefore, it is essential to identify and address potential risks in clinical practice. Second, probiotics and prebiotics often exhibit insufficient adhesion characteristics and short retention times in the gastrointestinal tract, thus leading to sub-optimal therapeutic results. This may result in extended treatment durations and diminished patient compliance due to the requirement for frequent supplementation (Xu et al., 2022). Future research should focus on improving the oral bioavailability and intestinal targeting efficacy of probiotics, possibly via the encapsulation of pharmaceuticals in nanoparticles (Xu et al., 2022). Furthermore, most studies relating to the health impacts of probiotics and prebiotics via the gut-brain axis have utilized animal models. The interactions and mechanisms between probiotics and human gut microbiota have yet to be investigated. Consequently, further studies necessitate more stringent and well-organized trials, the establishment of suitable animal models, and the formulation of tailored probiotic therapies. Recognizing potential drug interactions could enhance treatment results and minimize patient risks.

### Engineered bacteria

Engineering the design of microorganisms allows for the introduction of new functionality and the modification of existing functions; for example, engineered bacteria can exhibit reduced immunogenicity and enhanced targeting abilities, thus making them more useful (Fan et al., 2022). Little is known about the potential for engineered bacteria to treat neurodegenerative diseases, although there are a number of pathways by which these could be advantageous. To modulate the immune response in neurodegenerative diseases, engineered bacteria could utilize their own immunogenicity or act as delivery vehicles for immunostimulatory molecules, such as cytokines. Furthermore, engineered bacteria have the ability to transport therapeutic drugs, proteins, and small molecules directly to specific locations. Mitochondrial dysfunction is one of the primary causes for the most prevalent neurodegenerative diseases, including Huntington’s disease, PD and AD (Keethedeth and Anantha Shenoi, 2024). Consequently, engineered bacteria could be employed to enhance the delivery of mitochondrial therapeutics, including mtDNA, and to facilitate mitochondrial transplantation to alleviate the symptoms of disease. However, the clinical translation of engineered bacteria also faces certain challenges, including the absence of a specific mechanism for engineered bacterial therapy, an undefined dose-response relationship for live bacteria, the potential induction of immune-related adverse reactions, and unclear product stability. Therefore, successful clinical translation necessitates a comprehensive evaluation of the primary mechanisms of engineered bacteria, the establishment of the optimal bacterial dose, the meticulous assessment of potential risks and the reinforcement of close collaboration with regulatory agencies (Kwon et al., 2024).

### Fecal microbiota transplantation

Fecal microbiota transplantation (FMT) has achieved promising results for the treatment of neurodegenerative diseases by re-establishing a balanced intestinal microbiota community. There is some evidence that FMT can temporarily enhance gastrointestinal function and alleviate motor symptoms in individuals with PD (Huang et al., 2019). For example, a patient with AD exhibited increased cognitive scores and memory retention after receiving a single fecal microbiota transplant from his wife (Hazan, 2020). Conversely, germ-free wild-type mice transplanted with the intestinal microbes of AD patients presented with fewer nervous system-related metabolites and diminished cognitive function (Fujii et al., 2019). Interestingly, the underlying mechanism of FMT for the treatment of neurodegenerative diseases may be related to its effect on the microbiota–mitochondria crosstalk. FMT and SCFA supplementation could prevent oxidative phosphorylation (OXPHOS) dysfunction via mitochondrial metabolic reprogramming, thereby alleviating cognitive impairment by inhibiting chronic cerebral hypoperfusion (Su et al., 2023). FMT from healthy humans to MPTP-induced PD mice led to the inhibition of microgliosis and astrogliosis; this could ameliorate mitochondrial impairments via the AMPK/superoxide dismutase 2 pathway to restore blood–brain barrier integrity and the loss of nigrostriatal pericytes. These findings suggest that FMT can alleviate the symptoms of neurodegenerative diseases by correcting the imbalance of intestinal microorganisms to prevent mitochondrial dysfunction (Xie et al., 2023).

Nevertheless, there is substantial apprehension relating to the safety of FMT. The risk of diseases associated with gastrointestinal microbiome composition may be elevated as a result of the transmission of antibiotic-resistant bacteria and potentially harmful microorganisms by FMT (DeFilipp et al., 2019; Hill, 2020). Consequently, it is imperative to improve the screening of donors and monitor adverse infection events that are caused by anomalous microorganisms in clinical practice. Furthermore, the long-term safety and durability of the clinical efficacy of FMT is challenging to ascertain due to the relatively brief follow-up periods of the majority of clinical trials (8 to 12 weeks). Consequently, the follow-up period for patients must be extended in future clinical studies (Yadegar et al., 2024).

### Postbiotic therapy

A postbiotic is defined as a “preparation of inanimate microorganisms and/or their components that confers a health benefit on the host” (Salminen et al., 2021) according to the International Scientific Association of Probiotics and Prebiotics (ISAPP) agreement. Postbiotics, which mimic probiotic effects without needing specific storage, shipping, or production conditions, provide hope for the treatment and prevention of several neurodegenerative disorders (Sorbara and Pamer, 2022).

Because of their protective function in neurodegenerative diseases, SCFAs represent the most commonly studied of all microbiota metabolites. The role of butyrate in particular has been the subject of much research (Chakraborty et al., 2024). Researchers have demonstrated that sodium butyrate can restore the MMP of astrocytes to improve mitochondrial function and promote astrocyte differentiation into the A2 subtype, which can alleviate the symptoms of AD (Wang et al., 2022a). In addition, the upregulation of PGC1-α transcription, which increases the expression of important components of the mitochondrial electron transport chain, can improve mitochondrial respiration in models of neuronal ALS (Li et al., 2022; Karbownik et al., 2023). In addition, propionate and other SCFAs can alleviate brain atrophy and the progression of MS by enhancing regulatory T cell mitochondrial morphology and functionality (Duscha et al., 2020). One possible approach for the treatment of neurodegenerative diseases could be to monitor the levels of SCFAs in the gut microbiome and then supplement or control these levels when needed. The ability of mass spectrometry to detect and analyze metabolites and concentrations from various gut microbiota makes this a potentially useful tool (Han et al., 2015). Researchers have discovered that ALS patients exhibit lower levels of butyrate (Unger et al., 2016); this led to the quantitative analysis of SCFA levels by gas chromatography in the fecal samples of PD patients (Nicholson et al., 2021). Butyrate supplementation appears to delay the onset of ALS in mice (Zhang et al., 2017). Nevertheless, postbiotic treatment is still relatively new; consequently, there is a lack of data relating to benefits, dangers, and adverse effects. Therefore, further research is needed to ascertain the impact of metabolites and products on the CNS, both in a positive and negative manner. It is also important to carefully consider the specific concentrations utilized in this approach.

### Diet modification

A nutritious diet is crucial for sustaining a healthy neurological system across the lifespan by promoting a favorable gut microbiota composition. The ketogenic diet is a low-carbohydrate and high-fat dietary plan that relies on the generation of ketones, which serve as a more efficient energy source for brain cells (neurons and astrocytes) compared to glucose. Conversely, an alternative perspective posits that the medium-chain fatty acids in the ketogenic diet, rather than ketones, exert a neuroprotective effect by directly inhibiting AMPA receptors (glutamate receptors) and by modifying cellular energy via the regulation of mitochondrial biogenesis (Augustin et al., 2018). Clinical studies indicate that a modified ketogenic diet can enhance daily functionality and the quality of life of patients with AD (Phillips et al., 2021). Adhering to a low-fat or ketogenic diet can enhance both motor and non-motor symptoms in patients with PD, with a ketogenic cohort demonstrating superior outcomes in a previous study (Phillips et al., 2018). Researchers recently reported that a low-protein and high-carbohydrate diet provided a neuroprotective effect for a mouse model of MPTP-induced PD by modulating the microbiota-brain axis and fibroblast growth factor 21 (Chu et al., 2023). In addition, polyphenols have been shown to modify the composition and functionality of the intestinal microbiota and are transformed into bioavailable active phenolic metabolites by gut microbes. These phenolic metabolites traverse the blood–brain barrier, subsequently modulating mitochondrial function and mitigating oxidative stress to elicit neuroprotective effects (Rivas et al., 2022). For example, protocatechuic acid has the potential to increase health and lifespan, improve mitochondrial function, and contribute to healthy aging (Dilberger et al., 2019). The dietary polyphenol resveratrol has been shown not only to restore mitochondrial function by activating the SIRT1/PGC-1α signaling pathway (Lagouge et al., 2006) but also to protect cultured neocortical neurons acquired from mouse strains exhibiting accelerated-aging mouse strains from increased susceptibility to oxidative damage (Cristòfol et al., 2012).

Nevertheless, the gut microbiome, which can be influenced by diet and affect host metabolism, exhibits a highly intricate relationship that is characterized by substantial inter-individual variability. Thus, dietary therapy necessitates an extended duration and should be customized to individual conditions. Consequently, individualized dietary plans are gaining increasing significance. This methodology depends significantly on sophisticated technologies, including deep metagenomic sequencing of patient microbiomes, along with the acquisition of comprehensive long-term dietary data (Asnicar et al., 2021). Moreover, machine learning models can be utilized to investigate the composition and function of a patient’s microbiome, along with other host-related factors, to produce tailored dietary recommendations (Popp et al., 2022).

### Other drugs

Several other drugs have been found to exert neuroprotective effects by modulating gut microbiota–mitochondria crosstalk. For example, the mangosteen pericarp is an antioxidant-rich product that can alleviate motor deficits in PD by remodeling the composition of the fecal microbiota and by restoring mitochondrial function (Nurrahma et al., 2022). Recently, acarbose was shown to restructure the gut microbiota and alter the production of SCFAs to slow the progression of mitochondrial disease. Acarbose strongly increased the concentration of butyric acid which alleviated the symptoms of a mouse model of ALS by improving mitochondrial function (Zhang et al., 2017; Bitto et al., 2023).

Although there are some symptoms of neurodegenerative diseases that can be effectively treated with a single medication, such as memory loss, no such treatment has been able to prevent or cure a neurodegenerative disease as of yet. Treatment strategies that optimize the support and functionality of the mitochondrial network should take into account multiple factors, such as those related to nutrition, sleep, exercise, stress, and environmental factors since these all play a role in the onset of neurodegenerative diseases. Therefore, it is possible that a multi-therapeutic strategy that alters numerous underlying components of the mitochondrial network simultaneously will be more effective than a mono-therapeutic strategy (Rao et al., 2023).

## Conclusion and Prospect

In recent years, there has been a surge of interest in the gut–brain axis and the role of gut microbiota in the development and pathology of neurodegenerative diseases; however, the mechanisms by which this effect occurs are still unknown. It is now believed that mitochondria are very important mediators in the gut–brain axis. Important signs of neurodegenerative diseases include neuroinflammation and mitochondrial dysfunction. To understand how gut microbiota influence the development of neurodegenerative diseases, it is essential to study the interaction between gut microbiota, mitochondrial function, and neuroinflammation in the CNS. Neurodegenerative diseases such as AD, PD, and ALS are the primary focus of this review, which methodically and thoroughly summarizes the derivatives of gut microbes that control brain mitochondrial function, explains how gut microbes and their derivatives regulate central mitochondrial function and looks at the effects of targeting mitochondria to modulate the central immune system in this context. Additionally, we outlined the current and past approaches to treating neurodegenerative diseases by modifying the interactions between the intestinal microbiota and mitochondria. These approaches include the use of antibiotics, probiotics, prebiotics, engineered bacteria, postbiotic therapy, dietary changes, and FMT. In conclusion, intestinal microbiota-mitochondrial interaction plays a crucial role in the occurrence and development of neurodegenerative diseases, and there may be a way to cure these diseases in the future by targeting these interactions in therapeutic tactics. However, the interaction between the gut microbiota and mitochondria is currently a relatively underexplored aspect of neurodegenerative diseases, and it has been very poorly studied in neurodegenerative diseases other than AD, PD, and ALS. Therefore, the review lacks a comprehensive summary of the effect of the gut microbiota-mitochondria crosstalk on other neurodegenerative diseases, such as Huntington’s disease and multiple sclerosis.

In the future, it is imperative to conduct additional research on the importance of intestinal microbiota-mitochondrial crosstalk in other neurodegenerative diseases, including Huntington’s disease and multiple sclerosis. Furthermore, further research is required to clarify the mechanism of specific intestinal microbial-derived substances, including SCFAs, which have been shown to have a unique effect on neurodegenerative diseases. Furthermore, research has shown that IPA is involved in the regeneration and repair of nerves (Serger et al., 2022). However, its research on neurodegenerative diseases remains restricted. It is imperative to ascertain which patient populations could benefit from a specific treatment strategy, as the potential strategies that have been developed thus far are not effective for all patients. Therefore, the way forward is to create personalized treatment plans that are influenced by each individual’s unique gut microbiota, among other variables. The analysis of the composition of intestinal microorganisms (taxonomy and biodiversity), microbiome genes, and functional microorganisms, as well as the potential relationship between the microbiome and disease risk, has become feasible with the widespread application of sequencing technologies such as 16S rRNA sequencing, shotgun metagenomic sequencing (for total DNA), and RNA sequencing (Zhao et al., 2021a; Jin et al., 2022). As a result, these technologies can be integrated with genetic engineering and nanotechnology to create engineered bacteria that are tailored to the patient’s intestinal microbiota and genes, thereby enabling the treatment of diseases that are both personalized and targeted. Nevertheless, the translation of laboratory research into clinical trials is a complex process, and preclinical studies and clinical trials utilizing intestinal microbiota to treat neurodegenerative diseases are still in the early stages. Although animal experiments can offer some insight into the characteristics and functions of specific intestinal microbial communities, there are substantial disparities between the gut microbiomes of humans and animals. Consequently, personalized gut microbiome treatments necessitate meticulous design and extensive, iterative clinical trials.

## Additional files:

***Additional Table 1:*** *Summary of the mechanism and effect of derivates from gut microbiota on CNS mitochondria during neurodegenerative disorders.*

***Additional Table 2:*** *Summary of treatment approaches for neurodegenerative diseases that target the microbiota‒mitochondria crosstalk.*

## Data Availability

*All relevant data are within the paper and its Additional files*.
